# Incentive strategy of dual innovation balance in green manufacturing innovation ecosystem: Based on hierarchical structure of innovation subject

**DOI:** 10.1371/journal.pone.0291811

**Published:** 2023-09-21

**Authors:** Hao Qin, Hua Zou, Jian Sun

**Affiliations:** 1 School of Management, Shenyang University of Technology, Shenyang, China; 2 School of Economics, Shenyang University of Technology, Shenyang, China; Hanyang University - Seoul Campus: Hanyang University, REPUBLIC OF KOREA

## Abstract

Exploratory innovation is a pivotal way to seize future opportunities in green manufacturing innovation ecosystems, and exploitative innovation is conducive to expanding existing markets and resources, so it is essential to discuss the balanced incentive strategy of dual innovation for the sustainable development of ecosystems. Based on the hierarchical structure, this paper divides the core subjects in the green manufacturing innovation ecosystem into the application layer, the support layer, and the scientific research layer, constructs the differential game model of the no-incentive scenario, the cost-sharing scenario, and the collaborative scenario, and discusses the incentive strategies of the three types of subjects and the ecosystem in the evolution process of the dual innovation balance. The conclusions are as follows: (1) The level of dual innovation balance effort of the three types of subjects decreases with the increase of resource costs and environmental construction costs and increases with the increase of innovation balance capacity; (2) Cost sharing from the application layer to the support layer and the scientific research layer can enhance the effort level of both, which in turn enhances the optimal benefits for the three types of subjects and the ecosystem as a whole; (3) In the collaborative scenario, the level of effort and total ecosystem benefits of the innovation balance of the three types of subjects are strictly better than in the no incentive scenario, and the Pareto-optimality of the three subjects and the ecosystem will be realized after the coefficients of the distribution of benefits among the three types of subjects are determined. Based on this, this paper puts forward specific suggestions for the optimization of the structural relationship of the innovation body hierarchy, the exploitation of green manufacturing resources, and the macro-planning of the management department.

## Introduction

Green manufacturing is a kind of advanced manufacturing mode in harmony and symbiosis with ecological civilization, integrating green processes and green concepts into the whole industrial chain of manufacturing industry and pursuing to realize the lowest environmental impact and the highest efficiency of resource exploitation across the entire life cycle of designing, manufacturing, packaging, transporting, using, and disposing of at the end of the life cycle [1]. Especially in the context of the changing climate environment, the development of green manufacturing is a common choice of significant economies around the world, and green manufacturing has also become a direct direction for the action of manufacturing science and technology and industrial change. In the specific operation process of green manufacturing, enterprises actively implement green manufacturing technology and produce green products, which directly promote the development of green manufacturing. That is to say, enterprises are the primary green manufacturing body with the core leading role. In the increasingly complex and ambiguous situation of global science and technology innovation, a separate innovation body can only obtain some of the resources needed for creation. The innovation ecosystem can significantly enhance the innovation ability of enterprises and improve their competitive advantage in the market [2]. BYD and other new energy vehicle enterprises have constructed an innovation ecosystem to enhance the innovation ability of green manufacturing technology. The green manufacturing innovation ecosystem provides an emerging paradigm for developing green manufacturing with the characteristics of green manufacturing and innovation ecosystems, realizing collaborative innovation of diversified subjects while deepening the connection between innovation subjects, resources, and the environment.

However, scholars have yet to reach a broad consensus on balancing the dual innovation of exploration and exploitation in the green manufacturing innovation ecosystem to realize the response of the innovators to the changes in the green innovation environment and promote the ecosystem’s sustainable development. Under the trend of increasing competition in the green manufacturing innovation environment, enterprises must play a leading role in the dual innovation balance, actively explore future market opportunities and develop new technological services, as well as expand their existing knowledge and skills [3], that is, to realize the balance between exploratory and exploitative dual innovation. An enterprise can fall into the failure trap if it emphasizes only one type of innovation activity [4]. In management practice, dual innovation balance is highly valued by system managers and governors, in which advanced innovation ecosystems such as Silicon Valley have strategically planned the classification of exploratory and exploitative innovation indicators. As a practical innovation approach in the green manufacturing innovation ecosystem, the Dual innovation balance has recently received extensive attention from scholars. Dodgson et al. [5] and Enkel et al. [6] proposed that using the existing resources and continuously exploring new opportunities will significantly improve the main body of the innovation capacity, and this process needs to grasp the use of external knowledge and resources. In the evolutionary process of dual innovation balance, scholars have paid attention to the central role of resources but still pay less attention to innovation infrastructure, green manufacturing governance, and other innovation environments, which have an essential macro-regulatory part in actual practice. Moreover, the innovation subject in the ecosystem is the action object of the dual innovation balance, with core functions such as resource acquisition, exploitation, and environment construction. Zhang et al. proposed that enterprises need to strengthen the linkage relationship with the government, colleges and universities, research institutions, intermediaries, and other subjects, increasing the openness of the green manufacturing system [7]. Shao et al. further emphasized that the overall collaborative relationship between multiple innovation subjects, the innovation factors, and the innovation environment promotes the stable and sustainable development of the innovation ecosystem [8]. The continuous optimization and improvement of the synergistic system of innovation main body, environment, and elements promotes the sustainable development of the green manufacturing innovation ecosystem, providing theoretical guidance for the realistic scenario. Incentive strategies to realize we should explore the dual innovation balance of the green manufacturing innovation ecosystem promptly and adequately. Combining green manufacturing and innovation ecosystems presents challenges and opportunities for traditional research. However, there are still fewer explorations based on the dual innovation balance in the context of green manufacturing, which highlights the urgent need for research on the dual innovation balance in green manufacturing innovation ecosystems. Further, this paper raises the following scientific questions: (1) What functions do the diversified innovation subjects in the green manufacturing innovation ecosystem play in realizing the dual innovation balance? And how is the ecosystem hierarchical structure divided according to the functions of the subjects? (2) How do different dual innovation balance scenarios based on the hierarchical structure realize the optimal benefits for innovation subjects and ecosystems? (3) Do variables such as resource costs, environmental construction costs, exploratory innovation costs, and exploitation innovation costs affect incentive strategies?

This paper adopts a hierarchical structure research framework to explore the dual innovation balanced incentive strategy of exploratory and exploitative green manufacturing innovation ecosystem. The research in this paper covers the linkage relationship and functional characteristics of ecosystem innovation subjects and the influence of both innovation environment and innovation resource elements on decision-making. Theoretically, the green manufacturing and innovation ecosystem dual innovation is refined to micro-level enterprises. This paper provides new practical support for optimizing the technological innovation approach in the green manufacturing industry.

## Theoretical basis and literature review

### Green manufacturing innovation ecosystem

Green manufacturing technology innovation is a complex technology and market process. The innovation process relies on mutual communication and integration between multiple subjects [9], and the innovation ecosystem highlights the synergistic innovation between multiple issues to realize value creation [10]. The green manufacturing innovation ecosystem integrates the typical characteristics of the green manufacturing and innovation ecosystem, emphasizes green manufacturing technology breakthroughs and multi-principal synergistic technological innovation, and is forward-looking in the development process of the green manufacturing field.

Scholars first research green manufacturing from a system perspective. The mainstream hotspot at this stage is the green manufacturing system. Zheng et al. defined the green manufacturing system as green energy, green production process, and green products with three core functional components, and the basis of the operational cycle of the green manufacturing system proved that there is a close relationship with the industrial ecosystem and the recycling of social systems [11]. The interaction between different subjects in the green manufacturing system increases the internal complexity of the system, making the relatively complex closed system transformed into a more complex open system, as Zhang et al., based on the development of China’s green manufacturing situation, proposed that the closed green manufacturing system consists of the green manufacturing enterprises, the government, colleges and universities, scientific research institutions and intermediary institutions and other central bodies. Still, for the open green manufacturing system, the main body of the innovation is further supplemented [7].

In recent years, the green manufacturing innovation ecosystem has become an innovation paradigm and a new industry. Cao et al. designed an industrial technology ecosystem by combining the principles of ecological species symbiosis and material cycling, transformation, and regeneration. They emphasized that the innovation model, product evaluation, and manufacturing process in green manufacturing belong to the system’s core [12]. Shao et al., on the other hand, proposed that the open innovation model of green manufacturing innovation ecosystem can enhance the efficiency of technological innovation, emphasizing that the system of elements in the system and the environmental role of the mechanism is an essential guarantee for the stable development of the system [8]. Factor flows in green manufacturing innovation ecosystems provide the kinetic energy for their product. Elisabeth, based on a study of U.S. manufacturing clusters, concluded that factor flows create pathways and opportunities for technological innovation capabilities based on the perspective of knowledge generation and flows within ecosystems [13].

### Exploratory innovation and exploitative innovation

Exploratory and exploitative innovation originated from the research of Western scholars March [14]; based on this classic theory, the essence of exploration is to try new possibilities, while utilization embodies the extension and improvement of existing capabilities, technologies, and paradigms. And then, it gradually came to the attention of Chinese scholars, among which Zhong et al. [15] were the first to conduct a study explicitly. A review of existing research reveals that the frontier areas of exploratory versus exploitative innovation focus on dynamic capabilities, organizational learning, technological innovation, strategic alliances, and organizational duality.

First, there is a strong link between dynamic capabilities and the balance and interaction between exploratory and exploitative innovations. Benner et al. [16] and Ancona et al. [17] believed that dynamic capabilities are derived from and rooted in exploratory and exploitative innovations. O’Reilly et al. proposed that dynamic capabilities are a whole that supports the development of exploratory and exploitative innovations from an integrated process perspective [18]. Whereas Kriz et al. equated exploratory and exploitative innovation as a dynamic capability [19], the realization of dynamic capability relies on variation and efficiency, where variation arises from exploration and efficiency arises from exploitation.

Secondly, organizational learning directly impacts firms’ exploratory and exploitative innovation. Dodgson concluded that corporate learning could explore new opportunities and maintain existing operations in unstable innovation environments to improve the subject’s innovation capability [5]. Benner et al. proposed the ensemble view to consider technological innovation as a subset of organizational learning [16], and therefore, organizational learning is a sufficient non-necessary condition for technological innovation.

Thirdly, the field of technological innovation applies and develops exploratory and exploitative innovation paradigms, and Enkel et al. found that external knowledge recognition capabilities significantly contribute to the development of exploratory and exploitative innovations. In contrast, absorptive capacities only affect exploratory innovations [6]. More studies have pointed out that national culture [20], leadership style [21], social networks [22], and knowledge heterogeneity [23] all influence the process of exploratory versus exploitative innovation evolution to varying degrees.

Fourthly, regarding the exploratory and exploitative innovation paradigm under which enterprises carry out technological innovation through strategic alliances, Koza et al. first introduced exploratory and exploitative innovations into the category of strategic partnerships. They used a dichotomous approach to categorize strategic alliances [24]. Then Rothaermel et al. suggested that incumbent firms can form exploitative and exploratory partnerships with new entrants to innovation in strategic alliances [25]. Krammer has extensively explored the effect of enterprise diversity, technological diversity, technological distance and product similarity on the heterogeneity generated by strategic alliances [26].

Fifth, organizational duality is a study of the balance and coordination of exploratory and exploitative innovations; He et al. earlier introduced the idea of the balance of exploratory and exploitative innovations into the connotation of the dual organization [27]. Duality is balancing exploratory and exploitation as the appropriate adaptive mechanism [28]. Still, balancing is not equal to compromise between the two, but instead optimizing the relationship between the two, and thus both perform well.

### Innovation balance

"Balance" belongs to the domain of mechanics theory in physics. When organizational innovation already has the power and elements, it is necessary to balance the complex network relationship between the choice of managerial innovation path and the construction of organizational structure [29]. Academics have carried out a wealth of research on innovation balance, mainly: First, the concept of innovation balance is defined, such as the balanced innovation main body (enterprises, universities and research institutions, research individuals) of science and technology innovation activities, but also includes the relevant government departments of the innovation services and management activities, but also scholars (consulting organizations), short for the innovation of the innovation research activities [30], and the innovation path evolution system within the enterprise is the process of balance-breaking the balance-new equilibrium [29]. Second, the choice of enterprise innovation balance model, including the dual innovation balance model of both exploratory and exploitative innovation [31], and the question of whether to choose intermittent or simultaneous equilibrium [32]. Third, the enhancement effect of enterprise innovation balance on enterprise performance, such as innovation balance can maintain both short-term and long-term development needs and maximize enterprise performance [33]. The knowledge search joint balance positively affects enterprise innovation performance [34]. Fourth, there are external influences on enterprises’ innovation balance, such as government subsidies that can effectively mitigate the impact of environmental uncertainty on the dual model [35], as well as innovation incentives and responsible innovation that directly influence the quality of innovation policies [36]. Fifth, the realization path of corporate innovation balance, such as the evolution law of corporate innovation balance and its evolution equilibrium state [37].

### Research gaps in existing literature

Throughout the relevant literature in the field of exploratory and exploitative dual innovation balance and incentive decision-making in green manufacturing innovation ecosystems, fruitful research results and perspectives have been developed, which provide a rich theoretical foundation and practical background for discovering the research questions in this paper. However, there are still the following research shortcomings: (1) For the study of green manufacturing technology innovation, the existing literature mainly focuses on one innovation mode or path and lacks an in-depth exploration of dual innovation, which may lead to an imbalance between the existing innovation process and future innovation opportunities for enterprises. (2) In the field of dual innovation research, the existing research perspectives have a low degree of articulation with the innovation ecosystem. As the scenario in which the innovation ecosystem is situated becomes more complex, the flow of resources becomes more frequent, the cooperation of subjects becomes more intense, and the innovation environment becomes more complicated, the introduction of the idea of dual innovation balance is helpful to understand the upgrading process of the innovation ecosystem in practice. (3) Existing literature is rich in research on the functions and characteristics of subjects in innovation ecosystems. Still, it lacks the delineation of the hierarchical structure of innovation subjects and lacks the incentive decision-making model of the hierarchical structure of innovation subjects, which limits the theoretical explanatory ability to a certain extent.

Therefore, based on the innovation ecosystem and dual innovation theory, this paper first divides the innovation subjects in the green manufacturing innovation ecosystem into three layers: application layer, support layer, and scientific research layer, and then constructs a differential game model to discuss the incentive decision-making problems in the three scenarios of no-incentive scenario, cost-sharing scenario, and collaborative cooperation scenario, to promote the establishment of an efficient technological upgrading path in the green manufacturing innovation ecosystem, get out of the bottleneck of innovation, and provide theoretical guidance and decision-making references for the realization of high-quality development.

## Problem description and variable explanation

**Fig 1:**
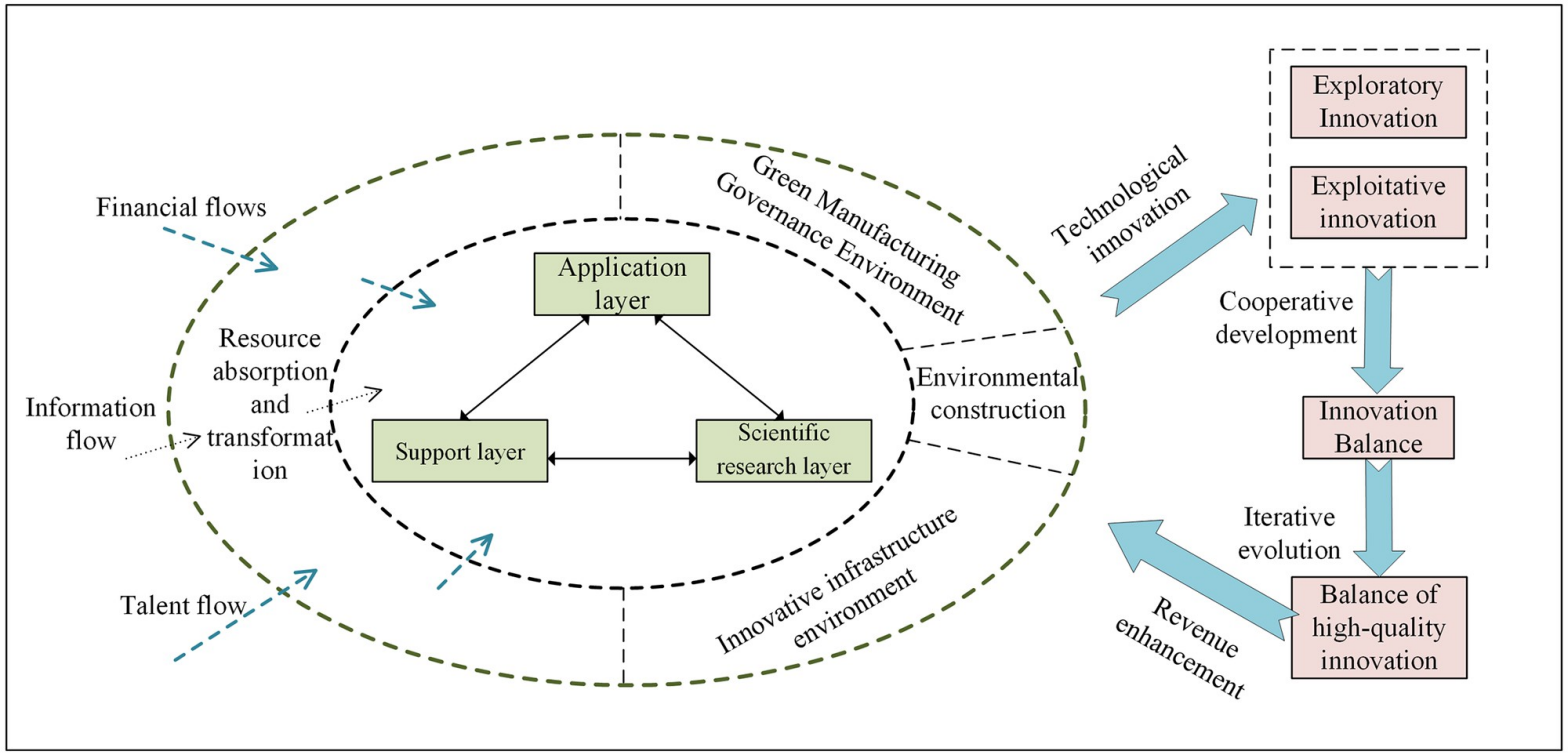
Logic of dual innovation balance in the green manufacturing innovation ecosystem.

The green manufacturing innovation ecosystem is a dynamic evolutionary complex system between different innovation subjects and innovation environments in the region relying on green technology to realize the combination of green innovation resources and non-green innovation resources to develop new products and services, with the characteristics of synergy, competition and symbiosis. Dual innovation is the optimal model for organizing technological innovation in an innovation ecosystem, and system governors need to balance exploratory and exploitative innovation to continuously promote the balance, complementarity and sustainability of system innovation [38]. Green manufacturing innovation ecosystem dual innovation balance is a dynamic process in which multiple subjects participate collaboratively, and this paper divides the innovation subjects into three layers: application layer, support layer, and scientific research layer, as shown in Fig 1.

Enterprises, as the core of the green manufacturing innovation ecosystem, are the main application subjects of green manufacturing technology, as well as the main body of R&D investment, innovation activities, results application and risk bearing, with the functions of guiding the creation and demand of technological innovation and promoting value co-creating and benefit distribution. The enterprise-led application layer effectively facilitates the diffusion and transfer of innovations in the ecosystem, as well as non-linear activities such as the productization of knowledge and technology through horizontal and vertical linkages with the prominent participants in the green supply chain, to bring into play the utility of the enterprise [39]. The support layer mainly includes the government, financial institutions, information intermediaries and other innovative entities, with core functions such as resource absorption and integration, innovation environment construction, introducing human resources and scientific research funds to the ecosystem, and building and optimizing environment of innovation technology facilities, green manufacturing governance, and institutional protection. The support layer is an essential driving body to ensure the balance of high-quality innovation in the green manufacturing innovation ecosystem, powerfully stimulate the creativity and dynamism of exploratory and utilization innovation, ensure the equal participation of all kinds of innovation subjects in the process of innovation balance, and then empower the innovation and creation vitality of micro issues. The scientific research layer, which mainly includes innovation subjects such as universities and research institutes, is the primary source of new knowledge generation [40], carries basic research and original innovation of green manufacturing technology, and promotes knowledge generation, transfer and integration of the green manufacturing innovation ecosystem, which energizes the process of its innovation balance.

**Fig 2:**
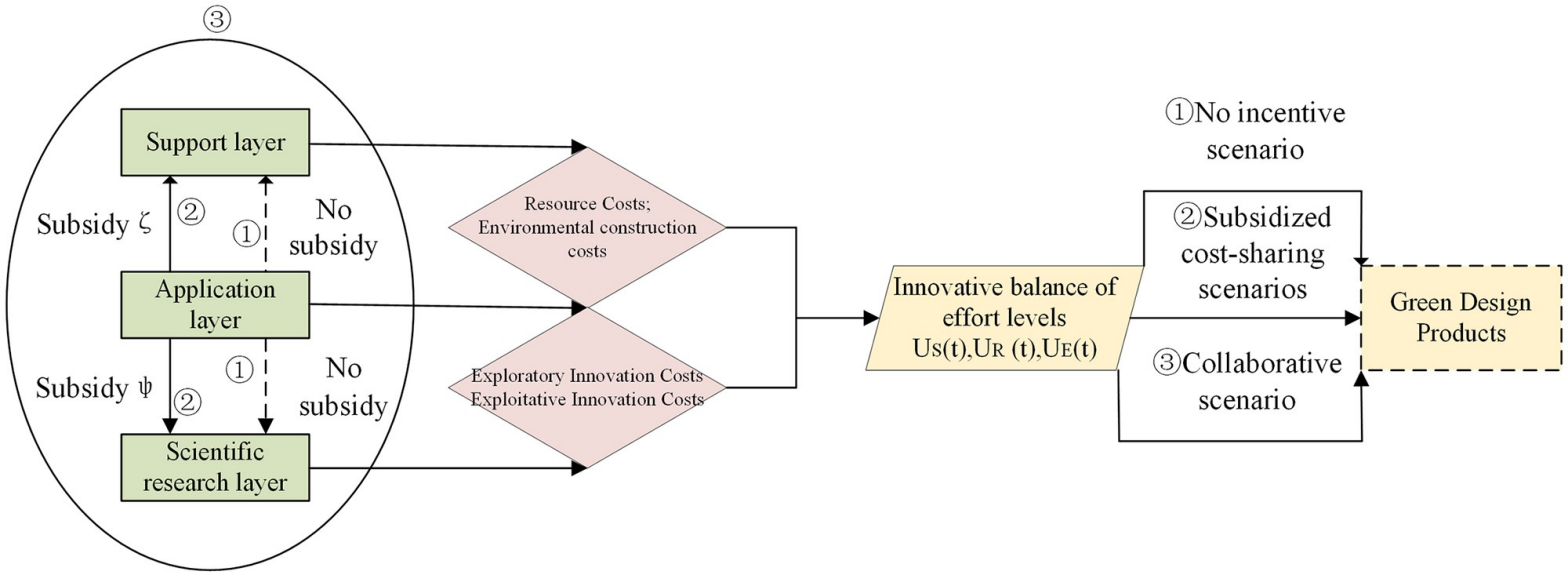
Dual innovation balance scenario with three types of subjects.

In the dynamic evolution of innovation balance of green manufacturing innovation ecosystem, combined with the reality of innovation, this paper divides the three types of innovation primary body cooperation into three scenarios, as shown in Fig 2. ① is the no-incentive scenario: the application layer does not subsidize the support layer and the scientific research layer, and the three types of innovation subjects make decisions independently without interfering with each other, all aiming at maximizing their revenue, which is in line with the Nash non-cooperative game. ② is the cost-sharing scenario: the application layer subsidizes the support layer and the scientific research layer in proportion *ζ* and *ψ*, respectively, in which the application layer takes action first, and the support layer and the scientific research layer follow to maximize their benefits and the ecosystem, which is in line with the Stackelberg game.③ is the collaborative cooperation scenario: the three types of innovation subjects collaborate in decision-making to maximize the benefits of the ecosystem, reflecting the characteristics of the ecosystem’s coupled symbiosis of technological innovation.

For ease of description, this paper begins by describing the symbols and their meanings, as shown in Table 1.

**Table 1:** Model symbols and meanings.

Symbols	Meaning
Decision variables	
*U*_*i*_(*t*), (*i* = *S*, *R*, *E*)	The level of innovative balance of effort of participating agents at moment t.
*ζ*	Balancing costs of innovation shared by the application layer for the support layer.
*ψ*	The balancing cost of innovation shared by the application layer for the scientific research layer.
Participant variables	
*ν*_*i*_, (*i* = *S*, *E*)	Resource cost factor, *ν*_*i*_ > 0
*μ*_*i*_, (*i* = *S*, *E*)	Environmental construction cost factor, *μ*_*i*_ > 0
*ω*_*i*_, (*i* = *R*, *E*)	Exploratory innovation cost factor, *ω*_*i*_ >0
*λ*_*i*_, (*i* = *R*, *E*)	Exploitative innovation cost factor, *λ*_*i*_ >0
State variables and game parameters	
*I*(*t*)	Level of innovation balance at moment t, *I*(0) = *I*_0_ ≥ 0
*γ*_*i*_, (*i* = *R*, *S*, *E*)	The extent to which the level of effort affects the total return to the system.
*η*_*i*_, (*i* = *R*, *S*, *E*)	Innovative balancing capacity factor.
*θ*	The extent to which the innovation balance affects the total return to the system.
*ε*	The natural decay rate of innovations in the technology development process.
*π*(*t*)	Total return function.
*V*_*i*_, (*i* = *R*, *S*, *E*)	Benefit Functions of participating subjects.
*α*	The return sharing factor for the support layer.
*β*	Earnings distribution coefficients for the scientific research layer.
*ρ*	Discount rate for future returns, 0 < *ρ* < 1

## A differential game model analysis of balanced incentive strategies for dual innovation

### Model assumptions

**Hypothesis 1:** The innovation balance cost of the support, research and application layers is related to the level of effort cost, and the innovation balance cost shows the law of diminishing marginal returns and is a convex function of the level of effort. That is, the higher the level of effort invested, the higher the innovation balance cost. The innovation balance cost of the green manufacturing innovation ecosystem mainly considers four factors: resource cost, environmental construction cost, exploratory innovation cost, and exploitative innovation cost. The cost of resources is $\frac{\nu_{i}}{2}U_{i}^{2}(t),(i=S,E)$, the cost of building the environment is $\frac{\mu_{i}}{2}U_{i}^{2}(t),(i=S,E)$, the cost of exploratory innovation is $\frac{\omega_{i}}{2}U_{i}^{2}(t),(i=R,E)$, and the cost of exploitative innovation is $\frac{\lambda_{i}}{2}U_{i}^{2}(t),(i=R,E)$. The innovation balance cost function of the participating subjects is shown in Eq (1).


$$\begin{array}{c} C_{S}(t)=\frac{\nu_{S}+\mu_{S}}{2}U_{S}^{2}(t);\\ C_{R}(t)=\frac{\omega_{R}+\lambda_{R}}{2}U_{R}^{2}(t);\\ C_{E}(t)=\frac{\nu_{E}+\mu_{E}+\omega_{E}+\lambda_{E}}{2}U_{E}^{2}(t) \end{array}$$ (1)

**Hypothesis 2:** The dual innovation balance between exploratory and exploitative is a dynamic process that requires participants to continuously deepen their cooperation and innovation to achieve optimal benefit distribution. In this paper, the innovation balance level is regarded as a state variable, which is affected by each participant’s effort level, support capacity, and R&D capacity. The innovation balance process of technological R&D innovation will naturally decay [41], assuming that the innovation balance level at the moment *t* is *I*(*t*), the innovation balance process of green manufacturing innovation ecosystem satisfies Eq (2).


$$I^{\prime }(t)=\eta_{E}U_{E}(t)+\eta_{S}U_{S}(t)+\eta_{R}U_{R}(t)-\varepsilon I(t)$$ (2)

**Hypothesis 3:** The total benefit function of the green manufacturing innovation ecosystem is shown in Eq (3).


$$\pi (t)=\gamma_{E}U_{E}(t)+\gamma_{S}U_{S}(t)+\gamma_{R}U_{R}(t)+\theta I(t)$$ (3)

**Hypothesis 4:** The support layer, the scientific research layer, and the application layer jointly distribute the total benefit of green manufacturing innovation balance, and the three participating subjects negotiate the benefit distribution ratio. As the dominant player in the innovation ecosystem, the application layer shares the technology development costs for the support layer and the scientific research layer, and the sharing ratio is regarded as the incentive coefficient of the application layer for the two innovation subjects. It is assumed that the same discount rate exists for the three types of innovation subjects, and all of them pursue the maximum return in the infinite time zone.

The objective function of the support layer is shown in Eq (4).


$$\text{max}\;J_{S}=\int_{0}^{\infty }e^{-\rho t}[\alpha \pi (t)+(\xi (t)-1)\frac{\nu_{S}+\mu_{S}}{2}U_{S}^{2}(t)]\text{d}\;t$$ (4)

The objective function of the scientific research layer is shown in Eq (5).


$$\text{max}\;J_{R}=\int_{0}^{\infty }e^{-\rho t}[\beta \pi (t)+(\psi (t)-1)\frac{\omega_{R}+\lambda_{R}}{2}U_{R}^{2}(t)]\text{d}\;t$$ (5)

The objective function of the application layer is shown in Eq (6).


$$\text{max}\;J_{E}=\int_{0}^{\infty }e^{-\rho t}[(1-\alpha -\beta )\pi (t)-\frac{\nu_{E}+\mu_{E}+\omega_{E}+\lambda_{E}}{2}U_{E}^{2}(t)-\xi (t)\frac{\nu_{S}+\mu_{S}}{2}U_{S}^{2}(t)-\psi (t)\frac{\omega_{R}+\lambda_{R}}{2}U_{R}^{2}(t)]\text{d}\;t$$ (6)

The differential game model constructed in this paper contains decision variables, participant variables, and state variables. Since the dynamic parameter conditions can-not be solved, assume all parameters are time-independent positive constants while omitting the time variable t. In the infinite time zone, the strategies of the three parties involved in the subject are the same, and drawing on the research of scholars [42], the strategies are regarded as static strategies, and the fixed feedback equilibrium solution is further obtained.

### Differential game modeling for three scenarios

#### No-incentive scenario

In the no-incentive scenario, the application layer does not provide any incentives to the support and scientific layers, that is, *ζ* = 0, *ψ* = 0. The three types of innovation subjects are balanced and uncooperative with each other, all aiming to maximize their benefits while choosing the most advantageous rational strategy. The optimal combination of methods for the green manufacturing innovation ecosystem in this scenario realizes the Nash equilibrium state.

The innovation return functions of all three types of innovation subjects are continuously bounded and differentiable and satisfy the HJB equation at *I* > 0, as in Eqs (7)–(9).


$$\rho V_{S}(I)={\text{max}}_{U_{S}\geq 0}\left\{\alpha \pi (t)-\frac{\nu_{S}+\mu_{S}}{2}U_{S}^{2}(t)+V_{S}^{\prime }(K)(\eta_{S}U_{S}+\eta_{R}U_{R}+\eta_{E}U_{E}-\varepsilon I)\right\}$$ (7)

$$\rho V_{R}(I)={\text{max}}_{U_{R}\geq 0}\left\{\beta \pi (t)-\frac{\omega_{R}+\lambda_{R}}{2}U_{R}^{2}(t)+V_{R}^{\prime }(K)(\eta_{S}U_{S}+\eta_{R}U_{R}+\eta_{E}U_{E}-\varepsilon I)\right\}$$ (8)

$$\rho V_{E}(I)={\text{max}}_{U_{E}\geq 0}\left\{\left(1-\alpha -\beta )\pi (t)-\frac{\nu_{E}+\mu_{E}+\omega_{E}+\lambda_{E}}{2}U_{E}^{2}(t)+V_{E}^{\prime }(K)(\eta_{S}U_{S}+\eta_{R}U_{R}+\eta_{E}U_{E}-\varepsilon I\right)\right\}$$ (9)

By taking the first-order partial derivatives of the right side of Eqs (7)–(9) for *U*_*S*_, *U*_*R*_, and *U*_*E*_, respectively and making them equal to 0, thus Eq (10) can be obtained.


$$U_{S}=\frac{\alpha \gamma_{S}+V_{S}^{\prime }(I)\eta_{M}}{\nu_{S}+\mu_{S}},U_{R}=\frac{\beta \gamma_{R}+V_{R}^{\prime }(I)\eta_{R}}{\omega_{R}+\lambda_{R}},U_{E}=\frac{(1-\alpha -\beta )\gamma_{E}+V_{E}^{\prime }(I)\eta_{E}}{\omega_{E}+\mu_{E}+\nu_{E}+\lambda_{E}}$$ (10)

Substituting Eq (10) into Eqs (7)–(9) simplifies to give Eqs (11)–(13).


$$\begin{array}{cc} \rho V_{S}(I) & =(\alpha \theta -\varepsilon V_{S}^{\prime }(I))I+\frac{{\left(\alpha \gamma_{S}+V_{S}^{\prime }\eta_{S}\right)}^{2}}{2(\nu_{S}+\mu_{S})}+\frac{\left(\beta \gamma_{R}+V_{R}^{\prime }\eta_{R})(\alpha \gamma_{R}+V_{S}^{\prime }\eta_{R}\right)}{\omega_{R}+\lambda_{R}}\\ & +\frac{\left[\gamma_{E}(1-\alpha -\beta )+V_{E}^{\prime }\eta_{E}](\alpha \gamma_{E}+V_{S}^{\prime }\eta_{E}\right)}{\omega_{E}+\mu_{E}+\nu_{E}+\lambda_{E}} \end{array}$$ (11)

$$\begin{array}{cc} \rho V_{R}(I) & =(\beta \theta -\varepsilon V_{S}^{\prime }(I))I+\frac{\left(\alpha \gamma_{S}+V_{S}^{\prime }\eta_{S})(\beta \gamma_{S}+V_{R}^{\prime }\eta_{S}\right)}{\nu_{S}+\mu_{S}}+\frac{{\left(\beta \gamma_{R}+V_{R}^{\prime }\eta_{R}\right)}^{2}}{2(\omega_{R}+\lambda_{R})}\\ & +\frac{\left[\gamma_{E}(1-\alpha -\beta )+V_{E}^{\prime }\eta_{E}](\beta \gamma_{E}+V_{R}^{\prime }\eta_{E}\right)}{\omega_{E}+\mu_{E}+\nu_{E}+\lambda_{E}} \end{array}$$ (12)

$$\begin{array}{cc} \rho V_{E}(I) & =[(1-\alpha -\beta )\theta -\varepsilon V_{S}^{\prime }(I)]I+\frac{\left[(1-\alpha -\beta )\gamma_{S}+V_{E}^{\prime }\eta_{S}](\alpha \gamma_{S}+V_{S}^{\prime }\eta_{S}\right)}{\nu_{S}}\\ & +\frac{\left[(1-\alpha -\beta )\gamma_{R}+V_{E}^{\prime }\eta_{S}](\beta \gamma_{R}+V_{R}^{\prime }\eta_{R}\right)}{\omega_{R}}+\frac{{\left[\gamma_{E}(1-\alpha -\beta )+V_{E}^{\prime }\eta_{E}\right]}^{2}}{2(\omega_{E}+\nu_{E})} \end{array}$$ (13)

Observing the form of Eqs (11)–(13), it is clear that the solution of the HJB equation is a one-variable functional equation with *I* as the independent variable, such that:

$$V_{S}(I)=k_{1}I+k_{2},V_{R}(I)=m_{1}I+m_{2},V_{E}(I)=n_{1}I+n_{2}$$ (14)

The factors in Eq (14) are constants to be solved for:

$$V_{S}{}^{\prime }(I)=\frac{dV_{S}(I)}{dI}=k_{1},V_{R}{}^{\prime }(I)=\frac{dV_{R}(I)}{dI}=m_{1},V_{E}{}^{\prime }(I)=\frac{dV_{E}(I)}{dI}=n_{1}$$ (15)

Substituting Eqs (14) and (15) into Eqs (11)–(13) gives:

$$\begin{array}{cc} \rho (k_{1}I+k_{2}) & =(\alpha \theta -\varepsilon k_{1})I+\frac{{\left(\alpha \gamma_{S}+k_{1}\eta_{S}\right)}^{2}}{2(\nu_{S}+\mu_{S})}+\frac{\left(\beta \gamma_{R}+m_{1}\eta_{R})(\alpha \gamma_{R}+k_{1}\eta_{R}\right)}{\omega_{R}+\lambda_{R}}\\ & +\frac{\left[\gamma_{E}(1-\alpha -\beta )+n_{1}\eta_{E}](\alpha \gamma_{E}+k_{1}\eta_{E}\right)}{\omega_{E}+\lambda_{E}+\mu_{E}+\nu_{E}} \end{array}$$ (16)

$$\begin{array}{cc} \rho (m_{1}I+m_{2}) & =(\beta \theta -\varepsilon k_{1})I+\frac{\left(\alpha \gamma_{S}+k_{1}\eta_{S})(\beta \gamma_{S}+m_{1}\eta_{S}\right)}{\nu_{S}+\mu_{S}}+\frac{{\left(\beta \gamma_{R}+m_{1}\eta_{R}\right)}^{2}}{2(\omega_{R}+\lambda_{R})}\\ & +\frac{\left[\gamma_{E}(1-\alpha -\beta )+n_{1}\eta_{E}](\beta \gamma_{E}+m_{1}\eta_{E}\right)}{\omega_{E}+\lambda_{E}+\nu_{E}+\mu_{E}} \end{array}$$ (17)

$$\begin{array}{cc} \rho (n_{1}I+n_{2}) & =[(1-\alpha -\beta )\theta -\varepsilon k_{1}]I+\frac{\left[(1-\alpha -\beta )\gamma_{S}+n_{1}\eta_{S}](\alpha \gamma_{S}+k_{1}\eta_{S}\right)}{\nu_{S}+\mu_{S}}\\ & +\frac{\left[(1-\alpha -\beta )\gamma_{R}+n_{1}\eta_{S}](\beta \gamma_{R}+m_{1}\eta_{R}\right)}{\omega_{R}+\lambda_{R}}+\frac{{\left[\gamma_{E}(1-\alpha -\beta )+n_{1}\eta_{E}\right]}^{2}}{2(\omega_{E}+\lambda_{E}+\nu_{E}+\mu_{E})} \end{array}$$ (18)

$$k_{1}=\frac{\alpha \theta }{\varepsilon +\rho },m_{1}=\frac{\beta \theta }{\varepsilon +\rho },n_{1}=\frac{(1-\alpha -\beta )\theta }{\varepsilon +\rho }$$ (19)

$$k_{2}=\frac{\alpha^{2}{\left[\gamma_{S}(\varepsilon +\rho )+\theta \eta_{S}\right]}^{2}}{2\rho (\nu_{S}+\mu_{S}){\left(\varepsilon +\rho \right)}^{2}}+\frac{\alpha \beta {\left[\gamma_{R}(\varepsilon +\rho )+\theta \eta_{R}\right]}^{2}}{\rho (\omega_{R}+\lambda_{R}){\left(\varepsilon +\rho \right)}^{2}}+\frac{\alpha (1-\alpha -\beta ){\left[\gamma_{E}(\varepsilon +\rho )+\theta \eta_{E}\right]}^{2}}{\rho (\omega_{E}+\lambda_{E}+\nu_{E}+\mu_{E}){\left(\varepsilon +\rho \right)}^{2}}$$ (20)

$$m_{2}=\frac{\alpha \beta {\left[\gamma_{S}(\varepsilon +\rho )+\theta \eta_{S}\right]}^{2}}{\rho (\nu_{S}+\mu_{S}){\left(\varepsilon +\rho \right)}^{2}}+\frac{\beta^{2}{\left[\gamma_{R}(\varepsilon +\rho )+\theta \eta_{R}\right]}^{2}}{2\rho (\omega_{R}+\lambda_{R}){\left(\varepsilon +\rho \right)}^{2}}+\frac{\beta (1-\alpha -\beta ){\left[\gamma_{E}(\varepsilon +\rho )+\theta \eta_{E}\right]}^{2}}{\rho (\omega_{E}+\lambda_{E}+\mu_{E}+\nu_{E}){\left(\varepsilon +\rho \right)}^{2}}$$ (21)

$$n_{2}=\frac{\alpha (1-\alpha -\beta ){\left[\gamma_{S}(\varepsilon +\rho )+\theta \eta_{S}\right]}^{2}}{\rho (\nu_{S}+\mu_{S}){\left(\varepsilon +\rho \right)}^{2}}+\frac{\beta (1-\alpha -\beta ){\left[\gamma_{R}(\varepsilon +\rho )+\theta \eta_{R}\right]}^{2}}{\rho (\omega_{R}+\lambda_{R}){\left(\varepsilon +\rho \right)}^{2}}+\frac{{\left(1-\alpha -\beta \right)}^{2}{\left[\gamma_{E}(\varepsilon +\rho )+\theta \eta_{E}\right]}^{2}}{2\rho (\omega_{E}+\lambda_{E}+\mu_{E}+\nu_{E}){\left(\varepsilon +\rho \right)}^{2}}$$ (22)

Substitute Eq (19) into Eq (10) to find the optimal level of effort of the three types of innovation subjects, as in Eq (23).


$$U_{S_{1}}^{*}=\frac{\alpha [\gamma_{S}(\varepsilon +\rho )+\theta \eta_{S}]}{\left(\nu_{S}+\mu_{S})(\varepsilon +\rho \right)},U_{R_{1}}^{*}=\frac{\beta [\gamma_{R}(\varepsilon +\rho )+\theta \eta_{R}]}{\left(\omega_{R}+\lambda_{R})(\varepsilon +\rho \right)},U_{E_{1}}^{*}=\frac{\left(1-\alpha -\beta )[\gamma_{E}(\varepsilon +\rho )+\theta \eta_{E}\right]}{\left(\nu_{E}+\mu_{E}+\omega_{E}+\lambda_{E})(\varepsilon +\rho \right)}$$ (23)

Substituting Eqs (19)–(22) into Eq (14), the optimal return functions for the three types of innovative subjects are obtained as Eqs (24)–(26).


$$\begin{array}{cc} V_{S1}{\left(I\right)}^{*} & =\frac{\alpha \theta }{\varepsilon +\rho }I+\frac{\alpha^{2}{\left[\gamma_{S}(\varepsilon +\rho )+\theta \eta_{S}\right]}^{2}}{2\rho (\nu_{S}+\mu_{S}){\left(\varepsilon +\rho \right)}^{2}}+\frac{\alpha \beta {\left[\gamma_{R}(\varepsilon +\rho )+\theta \eta_{R}\right]}^{2}}{\rho (\omega_{R}+\lambda_{R}){\left(\varepsilon +\rho \right)}^{2}}\\ & +\frac{\alpha (1-\alpha -\beta ){\left[\gamma_{E}(\varepsilon +\rho )+\theta \eta_{E}\right]}^{2}}{\rho (\omega_{E}+\lambda_{E}+\nu_{E}+\mu_{E}){\left(\varepsilon +\rho \right)}^{2}} \end{array}$$ (24)

$$\begin{array}{cc} V_{R1}{\left(I\right)}^{*} & =\frac{\beta \theta }{\varepsilon +\rho }I+\frac{\alpha \beta {\left[\gamma_{S}(\varepsilon +\rho )+\theta \eta_{S}\right]}^{2}}{\rho (\nu_{S}+\mu_{S}){\left(\varepsilon +\rho \right)}^{2}}+\frac{\beta^{2}{\left[\gamma_{R}(\varepsilon +\rho )+\theta \eta_{R}\right]}^{2}}{2\rho (\omega_{R}+\lambda_{R}){\left(\varepsilon +\rho \right)}^{2}}\\ & +\frac{\beta (1-\alpha -\beta ){\left[\gamma_{E}(\varepsilon +\rho )+\theta \eta_{E}\right]}^{2}}{\rho (\omega_{E}+\lambda_{E}+\nu_{E}+\mu_{E}){\left(\varepsilon +\rho \right)}^{2}} \end{array}$$ (25)

$$\begin{array}{cc} V_{E1}{\left(I\right)}^{*} & =\frac{(1-\alpha -\beta )\theta }{\varepsilon +\rho }I+\frac{\alpha (1-\alpha -\beta ){\left[\gamma_{S}(\varepsilon +\rho )+\theta \eta_{S}\right]}^{2}}{\rho (\nu_{S}+\mu_{S}){\left(\varepsilon +\rho \right)}^{2}}+\frac{\beta (1-\alpha -\beta ){\left[\gamma_{R}{\left(\varepsilon +\rho \right)}^{2}+\theta \eta_{R}\right]}^{2}}{\rho (\omega_{R}+\lambda_{R}){\left(\varepsilon +\rho \right)}^{2}}\\ & +\frac{{\left(1-\alpha -\beta \right)}^{2}{\left[\gamma_{E}(\varepsilon +\rho )+\theta \eta_{E}\right]}^{2}}{2\rho (\omega_{E}+\lambda_{E}+\nu_{E}+\mu_{E}){\left(\varepsilon +\rho \right)}^{2}} \end{array}$$ (26)

The total return of the green manufacturing innovation ecosystem is shown in Eq (27).


$$\begin{array}{cc} V{\left(I\right)}^{*} & =\frac{\theta }{\varepsilon +\rho }I+\frac{(2\alpha -\alpha^{2}){\left[\gamma_{S}(\varepsilon +\rho )+\theta \eta_{S}\right]}^{2}}{2\rho (\nu_{S}+\mu_{S}){\left(\varepsilon +\rho \right)}^{2}}+\frac{(2\beta -\beta^{2}){\left[\gamma_{R}(\varepsilon +\rho )+\theta \eta_{R}\right]}^{2}}{2\rho (\omega_{R}+\lambda_{R}){\left(\varepsilon +\rho \right)}^{2}}\\ & +\frac{[1-{\left(\alpha +\beta \right)}^{2}]{\left[\gamma_{E}(\varepsilon +\rho )+\theta \eta_{E}\right]}^{2}}{2(\omega_{E}+\lambda_{E}+\nu_{E}+\mu_{E})\rho {\left(\varepsilon +\rho \right)}^{2}} \end{array}$$ (27)

#### Cost-sharing scenario

In the cost-sharing scenario, the application layer is the dominant player in the green manufacturing innovation ecosystem, and the support and scientific research layers are the followers. The application layer subsidizes a certain percentage of the costs of the support and scientific research layers, which in turn increases their motivation to participate in the innovation balancing process. The application layer first determines the cost-subsidy ratio and effort level, and the support and scientific research layers, after observing the decisions of the application layer, will make corresponding follow-up strategies to maximize their benefits. At the same time, the application layer can predict the response strategies of the support and scientific research layers before making the final decision. Therefore, the scenario is consistent with the Stackelberg equilibrium and is solved by the backward induction method.

It is assumed that the optimal return functions of all three types of participating subjects are continuously bounded and differentiable and satisfy the HJB equation at *I* > 0, as in Eqs (28) and (29).


$$\rho V_{S}(I)={\text{max}}_{U_{S}\geq 0}\left\{\alpha \pi (t)+(\zeta -1)\frac{\nu_{S}+\mu_{S}}{2}U_{S}^{2}(t)+V_{M}^{\prime }(I)(\eta_{S}U_{S}+\eta_{R}U_{R}+\eta_{E}U_{E}-\varepsilon I)\right\}$$ (28)

$$\rho V_{R}(I)={\text{max}}_{U_{R}\geq 0}\left\{\beta \pi (t)+(\psi -1)\frac{\omega_{R}+\lambda_{R}}{2}U_{R}^{2}(t)+V_{R}^{\prime }(I)(\eta_{S}U_{S}+\eta_{R}U_{R}+\eta_{E}U_{E}-\varepsilon I)\right\}$$ (29)

Taking the right side of Eqs (28) and (29) to the first order partial derivatives of *U*_*S*_, *U*_*R*_ respectively, and making them equal to 0, then Eq (30) is obtained.


$$U_{S}=\frac{V_{S}^{\prime }(I)\eta_{S}+\alpha \gamma_{S}}{\left(1-\zeta )(\nu_{S}+\mu_{S}\right)},U_{R}=\frac{V_{R}^{\prime }(I)\gamma_{R}+\beta \gamma_{R}}{\left(1-\psi )(\omega_{R}+\lambda_{R}\right)}$$ (30)

The application layer decides its effort and the proportion of cost subsidy to other participating entities based on the decisions of the support and scientific research layers, and its HJB equation is shown in Eq (31).


$$\begin{array}{cc} \rho V_{E}(I) & ={\text{max}}_{U_{E}\geq 0}\{(1-\alpha -\beta )\pi (t)-\frac{\nu_{E}+\mu_{E}+\omega_{E}+\lambda_{E}}{2}U_{E}^{2}(t)-\zeta \frac{\nu_{S}+\mu_{S}}{2}\\ & -\psi \frac{\omega_{R}+\lambda_{R}}{2}+V_{E}^{\prime }(I)(\eta_{S}U_{S}+\eta_{R}U_{R}+\eta_{E}U_{E}-\varepsilon I)\} \end{array}$$ (31)

Substituting Eq (30) into Eq (31) and taking the first-order partial derivatives of the right side of Eq (31) with respect to *U*_*E*_, *ζ* and making it equal to 0, we obtain:

$$U_{E}=\frac{(1-\alpha -\beta )\gamma_{E}+\eta_{E}V_{E}^{\prime }}{\nu_{E}+\mu_{E}+\lambda_{E}+\omega_{E}}$$ (32)

$$\zeta =\frac{\gamma_{S}(2-3\alpha -2\beta )+\eta_{S}[2V_{E}^{\prime }-V_{S}^{\prime }(I)]}{\gamma_{S}(2-\alpha -2\beta )+\eta_{S}[2V_{E}^{\prime }+V_{S}^{\prime }(I)]}$$ (33)

$$\varphi =\frac{\gamma_{R}(2-2\alpha -3\beta )+\eta_{R}[2V_{E}^{\prime }-V_{R}^{\prime }(I)]}{\gamma_{R}(2-2\alpha -\beta )+\eta_{R}[2V_{E}^{\prime }+V_{R}^{\prime }(I)]}$$ (34)

Eqs (30) and (32)–(34) are substituted into the HJB equation and then simplified to give Eqs (35)–(37).


$$\begin{array}{cc} \rho V_{S}(I) & =[\alpha \theta -\varepsilon V_{S}^{\prime }(I)]I+\frac{\left(\alpha \gamma_{S}+V_{S}^{\prime }(I)\eta_{S})[\gamma_{S}(2-\alpha -2\beta )+\eta_{S}(2V_{E}^{\prime }(I)+V_{S}^{\prime }(I)\right]}{4(\nu_{S}+\mu_{S})}\\ & +\frac{\left(\alpha \gamma_{R}+V_{S}^{\prime }(I)\eta_{R})[\gamma_{R}(2-2\alpha -\beta )+\eta_{R}(2V_{E}^{\prime }(I)+V_{R}^{\prime }(I)\right]}{2(\omega_{R}+\lambda_{R})}+\frac{\left[\alpha \gamma_{E}+V_{S}^{\prime }(I)\eta_{E}][(1-\alpha -\beta )\gamma_{E}+\eta_{E}V_{E}^{\prime }\right]}{\nu_{E}+\mu_{E}+\omega_{E}+\lambda_{E}} \end{array}$$ (35)

$$\begin{array}{cc} \rho V_{R}(I) & =[\alpha \theta -\varepsilon V_{R}^{\prime }(I)]I+\frac{\left(\beta \gamma_{S}+V_{S}^{\prime }(I)\eta_{S})[\gamma_{S}(2-\alpha -2\beta )+\eta_{S}(2V_{E}^{\prime }(I)+V_{S}^{\prime }(I))\right]}{2(\nu_{S}+\mu_{S})}\\ & +\frac{\left(\beta \gamma_{R}+V_{R}^{\prime }(I)\eta_{R})[\gamma_{R}(2-2\alpha -\beta )+\eta_{R}(2V_{E}^{\prime }(I)+V_{R}^{\prime }(I))\right]}{2(\omega_{R}+\lambda_{R})}+\frac{\left[\beta \gamma_{E}+V_{S}^{\prime }(I)\eta_{E}][(1-\alpha -\beta )\gamma_{E}+\eta_{E}V_{E}^{\prime }\right]}{\nu_{E}+\mu_{E}+\lambda_{E}+\omega_{E}} \end{array}$$ (36)

$$\begin{array}{cc} \rho V_{E}(I) & =[(1-\alpha -\beta )\theta -\varepsilon V_{E}^{\prime }(I)]I+{\frac{\left[\gamma_{S}(2-\alpha -2\beta )+\eta_{S}(2V_{E}^{\prime }(I)+V_{S}^{\prime }(I))\right]}{8(\nu_{S}+\mu_{S})}}^{2}\\ & +{\frac{\left[\gamma_{R}(2-2\alpha -\beta )+\eta_{R}(2V_{E}^{\prime }(I)+V_{R}^{\prime }(I))\right]}{8(\omega_{R}+\lambda_{R})}}^{2}\text{+}\frac{{\left[(1-\alpha -\beta )\gamma_{E}+\eta_{E}V_{E}^{\prime }\right]}^{2}}{2(\nu_{E}+\mu_{E}+\omega_{E}+\lambda_{E})} \end{array}$$ (37)

Observation of Eqs (35)–(37) shows that the solution of the HJB equation is a one-variable functional equation with *I* as the independent variable, such that:

$$V_{S}(I)=k_{3}I+k_{4},V_{R}(I)=m_{3}I+m_{4},V_{E}(I)=n_{3}I+n_{4}$$ (38)

The factors of Eq (38) are constants to be solved for:

$$V_{S}{}^{\prime }(I)=\frac{dV_{S}(I)}{dI}=k_{3},V_{R}{}^{\prime }(I)=\frac{dV_{R}(I)}{dI}=m_{3},V_{E}{}^{\prime }(I)=\frac{dV_{E}(I)}{dI}=n_{3}$$ (39)

Substituting Eqs (38) and (39) into Eqs (35)–(37) gives:

$$\begin{array}{cc} \rho (k_{3}I+k_{4}) & =(\alpha \theta -\varepsilon k_{3})I+\frac{\left(\alpha \gamma_{S}+\eta_{S}k_{3})[\gamma_{S}(2-\alpha -2\beta )+\eta_{S}(2n_{3}+k_{3})\right]}{4(v_{S}+\mu_{S})}\\ & +\frac{\left(\alpha \gamma_{R}+\eta_{R}k_{3})[\gamma_{R}(2-2\alpha -\beta )+\eta_{R}(2n_{3}+m_{3})\right]}{2(\omega_{R}+\lambda_{R})}\text{+}\frac{\left(\alpha \gamma_{E}+\eta_{E}k_{3})[\gamma_{E}(1-\alpha -\beta )+\eta_{E}n_{3}\right]}{v_{E}+\mu_{E}+\lambda_{E}+\omega_{E}} \end{array}$$ (40)

$$\begin{array}{cc} \rho (m_{3}I+m_{4}) & =(\beta \theta -\varepsilon m_{3})I+\frac{\left(\beta \gamma_{S}+\eta_{S}m_{3})[\gamma_{S}(2-\alpha -2\beta )+\eta_{S}(2n_{3}+k_{3})\right]}{2(v_{S}+\mu_{S})}\\ & +\frac{\left(\beta \gamma_{R}+\eta_{R}m_{3})[\gamma_{R}(2-2\alpha -\beta )+\eta_{R}(2n_{3}+m_{3})\right]}{2(\omega_{R}+\lambda_{R})}\text{+}\frac{\left(\beta \gamma_{E}+\eta_{E}m_{3})[\gamma_{E}(1-\alpha -\beta )+\eta_{E}m_{3}\right]}{v_{E}+\mu_{E}+\lambda_{E}+\omega_{E}} \end{array}$$ (41)

$$\begin{array}{cc} \rho (n_{3}I+n_{4}) & =[(1-\alpha -\beta )\theta -\varepsilon n_{3}]I+\frac{{\left[\gamma_{S}(2-\alpha -2\beta )+\eta_{S}(2n_{3}+k_{3})\right]}^{2}}{8(v_{S}+\mu_{S})}\\ & +\frac{{\left[\gamma_{R}(2-2\alpha -\beta )+\eta_{R}(2n_{3}+m_{3})\right]}^{2}}{8(\omega_{R}+\lambda_{R})}\text{+}\frac{\left[\gamma_{E}(1-\alpha -\beta )+\eta_{E}n_{3}\right]}{2(v_{E}+\mu_{E}+\omega_{E}+\lambda_{E})} \end{array}$$ (42)

The solution is obtained by solving based on the previous assumptions:

$$k_{3}=\frac{\alpha \theta }{\rho +\varepsilon },m_{3}=\frac{\beta \theta }{\rho +\varepsilon },n_{3}=\frac{(1-\alpha -\beta )\theta }{\rho +\varepsilon }$$ (43)

$$\begin{array}{cc} k_{4} & =\frac{\alpha (2-\alpha -2\beta ){\left[\gamma_{S}(\rho +\varepsilon )+\theta \eta_{s}\right]}^{2}}{4\rho (\nu_{S}+\mu_{S}){\left(\rho +\varepsilon \right)}^{2}}+\frac{\alpha (2-\beta -2\alpha ){\left[\gamma_{R}(\rho +\varepsilon )+\theta \eta_{R}\right]}^{2}}{2\rho (\omega_{R}+\lambda_{R}){\left(\rho +\varepsilon \right)}^{2}}\\ & \text{+}\frac{\alpha (1-\alpha -\beta ){\left[\gamma_{E}(\rho +\varepsilon )+\theta \eta_{E}\right]}^{2}}{(\nu_{E}+\mu_{E}+\omega_{E}+\lambda_{E})\rho {\left(\rho +\varepsilon \right)}^{2}} \end{array}$$ (44)

$$\begin{array}{cc} m_{4} & =\frac{\beta (2-\alpha -2\beta ){\left[\gamma_{S}(\rho +\varepsilon )+\theta \eta_{s}\right]}^{2}}{2\rho (\nu_{S}+\mu_{S}){\left(\rho +\varepsilon \right)}^{2}}+\frac{\beta (2-\beta -2\alpha ){\left[\gamma_{R}(\rho +\varepsilon )+\theta \eta_{R}\right]}^{2}}{4\rho (\omega_{R}+\lambda_{R}){\left(\rho +\varepsilon \right)}^{2}}\\ & \text{+}\frac{\alpha (1-\alpha -\beta ){\left[\gamma_{E}(\rho +\varepsilon )+\theta \eta_{E}\right]}^{2}}{\rho (\nu_{E}+\mu_{E}+\omega_{E}+\lambda_{E}){\left(\rho +\varepsilon \right)}^{2}} \end{array}$$ (45)

$$\begin{array}{cc} n_{4} & =\frac{{\left(2-\alpha -2\beta \right)}^{2}{\left[\gamma_{S}(\rho +\varepsilon )+\theta \eta_{s}\right]}^{2}}{8\rho (\nu_{S}+\mu_{S}){\left(\rho +\varepsilon \right)}^{2}}+\frac{{\left(2-\beta -2\alpha \right)}^{2}{\left[\gamma_{R}(\rho +\varepsilon )+\theta \eta_{R}\right]}^{2}}{8\rho (\omega_{R}+\lambda_{R}){\left(\rho +\varepsilon \right)}^{2}}\\ & \text{+}\frac{{\left(1-\alpha -\beta \right)}^{2}{\left[\gamma_{E}(\rho +\varepsilon )+\theta \eta_{E}\right]}^{2}}{2\rho (\nu_{E}+\mu_{E}+\omega_{E}+\lambda_{E}){\left(\rho +\varepsilon \right)}^{2}} \end{array}$$ (46)

Substituting Eq (43) into Eqs (30) and (32)–(34), the optimal level of effort of the three types of innovation subjects and the optimal incentive coefficients of the application layer for the support layer and the scientific research layer can be obtained as Eqs (47)–(49).


$$\begin{array}{c} U_{S_{2}}^{*}=\frac{\left(2-\alpha -2\beta )[\gamma_{S}(\rho +\varepsilon )+\theta \eta_{s}\right]}{2(\nu_{S}+\mu_{S})(\rho +\varepsilon )},\\ U_{R_{2}}^{*}=\frac{\left(2-2\alpha -\beta )[\gamma_{R}(\rho +\varepsilon )+\theta \eta_{R}\right]}{2(\omega_{R}+\lambda_{R})(\rho +\varepsilon )},\\ U_{E_{2}}^{*}=\frac{\left(1-\alpha -\beta )[\gamma_{E}(\rho +\varepsilon )+\theta \eta_{E}\right]}{\left(\nu_{E}+\mu_{E}+\omega_{E}+\lambda_{E})(\rho +\varepsilon \right)} \end{array}$$ (47)

$$\zeta^{*}=\left\{\begin{array}{l} \frac{2-3\alpha -2\beta }{2-\alpha -2\beta },3\alpha +2\beta \leq 2\\ 0,3\alpha +2\beta >2 \end{array}\right.$$ (48)

$$\psi^{*}=\left\{\begin{array}{l} \frac{2-2\alpha -3\beta }{2-2\alpha -\beta },2\alpha +3\beta \leq 2\\ 0,2\alpha +3\beta >2 \end{array}\right.$$ (49)

Substituting Eqs (43)–(46) into Eq (38), the optimal return functions for the three types of innovative subjects can be obtained as Eqs (50)–(52).


$$\begin{array}{cc} V_{S_{2}}{\left(I\right)}^{*} & =\frac{\alpha \theta }{\rho +\varepsilon }I+\frac{\alpha (2-\alpha -2\beta ){\left[\gamma_{S}(\rho +\varepsilon )+\theta \eta_{s}\right]}^{2}}{4(\nu_{S}+\mu_{S})\rho {\left(\rho +\varepsilon \right)}^{2}}\\ & +\frac{\alpha (2-\beta -2\alpha ){\left[\gamma_{R}(\rho +\varepsilon )+\theta \eta_{R}\right]}^{2}}{2\rho (\omega_{R}+\lambda_{R}){\left(\rho +\varepsilon \right)}^{2}}+\frac{\alpha (1-\alpha -\beta ){\left[\gamma_{E}(\rho +\varepsilon )+\theta \eta_{E}\right]}^{2}}{\rho (\nu_{E}+\mu_{E}+\omega_{E}+\lambda_{E}){\left(\rho +\varepsilon \right)}^{2}} \end{array}$$ (50)

$$\begin{array}{cc} V_{R_{2}}{\left(I\right)}^{*} & =\frac{\beta \theta }{\rho +\varepsilon }I+\frac{\beta (2-\alpha -2\beta ){\left[\gamma_{S}(\rho +\varepsilon )+\theta \eta_{s}\right]}^{2}}{2\rho (\nu_{S}+\mu_{S}){\left(\rho +\varepsilon \right)}^{2}}\\ & +\frac{\beta (2-\beta -2\alpha ){\left[\gamma_{R}(\rho +\varepsilon )+\theta \eta_{R}\right]}^{2}}{4\rho (\omega_{R}+\lambda_{R}){\left(\rho +\varepsilon \right)}^{2}}\text{+}\frac{\alpha (1-\alpha -\beta ){\left[\gamma_{E}(\rho +\varepsilon )+\theta \eta_{E}\right]}^{2}}{\rho (\nu_{E}+\mu_{E}+\omega_{E}+\lambda_{E}){\left(\rho +\varepsilon \right)}^{2}} \end{array}$$ (51)

$$\begin{array}{cc} V_{E_{2}}{\left(I\right)}^{*} & =\frac{(1-\alpha -\beta )\theta }{\rho +\varepsilon }+\frac{{\left(2-\alpha -2\beta \right)}^{2}{\left[\gamma_{S}(\rho +\varepsilon )+\theta \eta_{s}\right]}^{2}}{8\rho (\nu_{S}+\mu_{S}){\left(\rho +\varepsilon \right)}^{2}}\\ & +\frac{{\left(2-\beta -2\alpha \right)}^{2}{\left[\gamma_{R}(\rho +\varepsilon )+\theta \eta_{R}\right]}^{2}}{8\rho (\omega_{R}+\lambda_{R}){\left(\rho +\varepsilon \right)}^{2}}\text{+}\frac{{\left(1-\alpha -\beta \right)}^{2}{\left[\gamma_{E}(\rho +\varepsilon )+\theta \eta_{E}\right]}^{2}}{2\rho (\nu_{E}+\mu_{E}+\omega_{E}+\lambda_{E}){\left(\rho +\varepsilon \right)}^{2}} \end{array}$$ (52)

The total return function of the green manufacturing innovation ecosystem is shown in Eq (53).


$$\begin{array}{cc} V_{2}{\left(I\right)}^{*} & =\frac{\theta }{\rho +\varepsilon }I+\frac{[4-{\left(\alpha +2\beta \right)}^{2}]{\left[\gamma_{S}(\rho +\varepsilon )+\theta \eta_{s}\right]}^{2}}{8\rho (\nu_{S}+\mu_{S}){\left(\rho +\varepsilon \right)}^{2}}\\ & +\frac{[4-{\left(2\alpha +\beta \right)}^{2}]{\left[\gamma_{R}(\rho +\varepsilon )+\theta \eta_{R}\right]}^{2}}{8\rho (\omega_{R}+\lambda_{R}){\left(\rho +\varepsilon \right)}^{2}}+\frac{[1-{\left(\alpha +\beta \right)}^{2}]{\left[\gamma_{E}(\rho +\varepsilon )+\theta \eta_{E}\right]}^{2}}{2\rho (\nu_{E}+\mu_{E}+\omega_{E}+\lambda_{E}){\left(\rho +\varepsilon \right)}^{2}} \end{array}$$ (53)

#### Collaborative scenario

Under the collaborative scenario, the application layer, support layer, and scientific research layer collaborate to promote the evolution of innovation balance, and all three types of subjects aim to maximize the benefits of the green manufacturing innovation ecosystem and jointly determine the optimal level of effort and the optimal function. The effort costs borne by the application layer to the support and scientific layers are internal system fund transfers with incentive coefficients *ξ*, *ψ*, and 0 ≤ *ξ*, *ψ* ≤ 0, respectively. At this point, the objective function of the green manufacturing innovation ecosystem is shown in Eq (54).


$${\text{max}}_{U_{S}(t),U_{R}(t),U_{E}(t)}J=\int_{0}^{\infty }e^{-\rho t}[\pi (t)-\frac{\nu_{S}+\mu_{S}}{2}U_{S}^{2}(t)-\frac{\omega_{R}+\lambda_{R}}{2}U_{R}^{2}(t)-\frac{\nu_{E}+\mu_{E}+\omega_{E}+\lambda_{E}}{2}U_{E}^{2}(t)]\text{d}\;t$$ (54)

It is assumed that an optimal function of the green manufacturing innovation ecosystem exists that is continuously bounded and differentiable and satisfies the HJB equation for all *I* ≥ 0 as in Eq (55).


$$\begin{array}{cc} \rho V(I) & ={\text{max}}_{U_{S}\geq 0,U_{R}\geq 0,U_{E}\geq 0}\{\pi (t)-\frac{\nu_{S}+\mu_{S}}{2}U_{S}^{2}(t)-\frac{\omega_{R}+\lambda_{R}}{2}U_{R}^{2}(t)\\ & -\frac{\nu_{E}+\mu_{E}+\omega_{E}+\lambda_{E}}{2}U_{E}^{2}(t)+V^{\prime }(I)(\eta_{S}U_{S}+\eta_{R}U_{R}+\eta_{E}U_{E}-\varepsilon I)\} \end{array}$$ (55)

Taking the first-order partial derivative of *U*_*S*_, *U*_*R*_, *U*_*E*_ on the right side of Eq (55) and making it equal to 0, we obtain Eq (56).


$$U_{S}=\frac{\gamma_{S}+V_{S}^{\prime }(I)\eta_{M}}{\nu_{S}+\mu_{S}},U_{R}=\frac{\gamma_{R}+V_{R}^{\prime }(I)\eta_{R}}{\omega_{R}+\lambda_{R}},U_{E}=\frac{\gamma_{E}+V_{E}^{\prime }(I)\eta_{E}}{\omega_{E}+\lambda_{E}+\nu_{E}+\mu_{E}}$$ (56)

Substituting Eq (56) into Eq (55) simplifies it:

$$\rho V(I)=[\theta -\varepsilon V^{\prime }(I)]I+\frac{{\left[\gamma_{S}+\eta_{S}V^{\prime }(I)\right]}^{2}}{2(\nu_{S}+\mu_{S})}+\frac{{\left[\gamma_{R}+\eta_{R}V^{\prime }(I)\right]}^{2}}{2(\omega_{R}+\lambda_{R})}+\frac{{\left[\gamma_{E}+\eta_{E}V^{\prime }(I)\right]}^{2}}{2(\omega_{E}+\lambda_{E}+\nu_{E}+\mu_{E})}$$ (57)

Observation of Eq (57) shows that the solution of the HJB equation is a one-variable functional equation with *I* as the independent variable, such that:

$$V(I)=k_{5}I+k_{6}$$ (58)

Where *K*_5_ and *K*_6_ are constants to be solved and solving yields Eq (59).


$$V(I)=\frac{dV(I)}{dI}=k_{5}$$ (59)

Substituting Eqs (58) and (59) into Eq (57) yields Eq (60).


$$\rho (k_{5}I+k_{6})=(\theta -\varepsilon k_{5})I+\frac{{\left(\alpha \gamma_{S}+k_{5}\eta_{S}\right)}^{2}}{2(\nu_{S}+\mu_{S})}+\frac{{\left(\gamma_{R}+k_{5}\eta_{R}\right)}^{2}}{2(\omega_{R}+\lambda_{R})}+\frac{{\left(\gamma_{E}+k_{5}\eta_{E}\right)}^{2}}{2(\omega_{E}+\lambda_{E}+\nu_{E}+\mu_{E})}$$ (60)

From the above assumptions, it can be seen that it is satisfied for all *I* ≥ 0. Thus, the values of *K*_5_, *K*_6_ can be found in Eqs (61) and (62).


$$k_{5}=\frac{\theta }{\rho +\varepsilon }$$ (61)

$$k_{6}=\frac{{\left[\gamma_{S}(\rho +\varepsilon )+\theta \eta_{S}\right]}^{2}}{2\rho (\nu_{S}+\mu_{S}){\left(\rho +\varepsilon \right)}^{2}}+\frac{{\left[\gamma_{R}(\rho +\varepsilon )+\theta \eta_{R}\right]}^{2}}{2\rho (\omega_{R}+\lambda_{R}){\left(\rho +\varepsilon \right)}^{2}}+\frac{{\left[\gamma_{E}(\rho +\varepsilon )+\theta \eta_{E}\right]}^{2}}{2\rho (\omega_{E}+\lambda_{E}+\nu_{E}+\mu_{E}){\left(\rho +\varepsilon \right)}^{2}}$$ (62)

Substituting Eq (61) into Eq (56), the optimal level of effort of the three types of innovation subjects is obtained as Eq (63).


$$U_{S_{3}}^{*}=\frac{\gamma_{S}(\rho +\varepsilon )+\theta \eta_{S}}{(\nu_{S}+\mu_{S}){\left(\rho +\varepsilon \right)}^{2}},U_{R_{3}}^{*}=\frac{\gamma_{R}(\rho +\varepsilon )+\theta \eta_{R}}{\left(\omega_{R}+\lambda_{R})(\rho +\varepsilon \right)},U_{E_{3}}^{*}=\frac{\gamma_{E}(\rho +\varepsilon )+\theta \eta_{E}}{\left(\omega_{E}+\lambda_{E}+\nu_{E}+\mu_{E})(\rho +\varepsilon \right)}$$ (63)

Substituting Eqs (61) and (62) into Eq (58), the optimal return function of green manufacturing innovation ecosystem is obtained as Eq (64).


$$V_{3}{\left(I\right)}^{*}=\frac{\theta }{\rho +\varepsilon }I+\frac{{\left[\gamma_{S}(\rho +\varepsilon )+\theta \eta_{S}\right]}^{2}}{2\rho (\nu_{S}+\mu_{S}){\left(\rho +\varepsilon \right)}^{2}}+\frac{{\left[\gamma_{R}(\rho +\varepsilon )+\theta \eta_{R}\right]}^{2}}{2\rho (\omega_{R}+\lambda_{R}){\left(\rho +\varepsilon \right)}^{2}}+\frac{{\left[\gamma_{E}(\rho +\varepsilon )+\theta \eta_{E}\right]}^{2}}{2\rho (\omega_{E}+\lambda_{E}+\nu_{E}+\mu_{E}){\left(\rho +\varepsilon \right)}^{2}}$$ (64)

The optimal return functions for the three types of innovative subjects are shown in Eqs (65)–(67).


$$V_{S_{3}}{\left(I\right)}^{*}=\frac{\alpha \theta }{\rho +\varepsilon }I+\frac{\alpha {\left[\gamma_{S}(\rho +\varepsilon )+\theta \eta_{S}\right]}^{2}}{2\rho (\nu_{S}+\mu_{S}){\left(\rho +\varepsilon \right)}^{2}}+\frac{\alpha {\left[\gamma_{R}(\rho +\varepsilon )+\theta \eta_{R}\right]}^{2}}{2\rho (\omega_{R}+\lambda_{R}){\left(\rho +\varepsilon \right)}^{2}}+\frac{\alpha {\left[\gamma_{E}(\rho +\varepsilon )+\theta \eta_{E}\right]}^{2}}{2\rho (\omega_{E}+\lambda_{E}+\nu_{E}+\mu_{E}){\left(\rho +\varepsilon \right)}^{2}}$$ (65)

$$V_{R_{3}}{\left(I\right)}^{*}=\frac{\beta \theta }{\rho +\varepsilon }I+\frac{\beta {\left[\gamma_{S}(\rho +\varepsilon )+\theta \eta_{S}\right]}^{2}}{2\rho (\nu_{S}+\mu_{S}){\left(\rho +\varepsilon \right)}^{2}}+\frac{\beta {\left[\gamma_{R}(\rho +\varepsilon )+\theta \eta_{R}\right]}^{2}}{2\rho (\omega_{R}+\lambda_{R}){\left(\rho +\varepsilon \right)}^{2}}+\frac{\beta {\left[\gamma_{E}(\rho +\varepsilon )+\theta \eta_{E}\right]}^{2}}{2\rho (\omega_{E}+\lambda_{E}+\nu_{E}+\mu_{E}){\left(\rho +\varepsilon \right)}^{2}}$$ (66)

$$\begin{array}{cc} V_{E_{3}}{\left(I\right)}^{*} & =\frac{(1-\alpha -\beta )\theta }{\rho +\varepsilon }I+\frac{(1-\alpha -\beta ){\left[\gamma_{S}(\rho +\varepsilon )+\theta \eta_{S}\right]}^{2}}{2\rho (\nu_{S}+\mu_{S}){\left(\rho +\varepsilon \right)}^{2}}\\ & +\frac{(1-\alpha -\beta ){\left[\gamma_{R}(\rho +\varepsilon )+\theta \eta_{R}\right]}^{2}}{2\rho (\omega_{R}+\lambda_{R}){\left(\rho +\varepsilon \right)}^{2}}+\frac{(1-\alpha -\beta ){\left[\gamma_{E}(\rho +\varepsilon )+\theta \eta_{E}\right]}^{2}}{2\rho (\omega_{E}+\lambda_{E}+\mu_{E}+\nu_{E}){\left(\rho +\varepsilon \right)}^{2}} \end{array}$$ (67)

### Comparative analysis of differential game modeling results

This paper draws the following conclusions by comparing the optimal effort level, the optimal benefit function of the green manufacturing innovation ecosystem for the support layer, the scientific research layer, and the application layer under the three scenarios.

**Proposition 1:** Compared with the no-incentive scenario, the application layer has the same level of effort in the cost-sharing scenario, and the effort levels of both the support layer and the scientific research layer are increased by a range equal to the proportion of the application layer that subsidizes their costs, that is the optimal incentive coefficient. The collaborative scenario has the highest level of effort for all three types of innovators, as shown in Eq (68).


$$\begin{array}{c} U_{S_{1}}^{*}<U_{S_{2}}^{*}<U_{S_{3}}^{*},U_{R_{1}}^{*}<U_{R_{2}}^{*}<U_{R_{3}}^{*},U_{E_{1}}^{*}=U_{E_{2}}^{*}<U_{E_{3}}^{*}\\ \frac{U_{S_{2}}^{*}-U_{S_{1}}^{*}}{U_{S_{2}}^{*}}=\zeta^{*}(0<\alpha <\frac{2}{3}),\frac{U_{R_{2}}^{*}-U_{R_{1}}^{*}}{U_{R_{2}}^{*}}=\varphi^{*}(0<\beta <\frac{2}{3}) \end{array}$$ (68)

**Proof**:

Eqs (23), (47)–(49), (63) lead to Eqs (69)–(73).


$$\begin{array}{cc} U_{S_{2}}^{*}-U_{S_{1}}^{*} & =\frac{\left(2-3\alpha -2\beta )[\gamma_{S}(\rho +\varepsilon )+\theta \eta_{S}\right]}{2(\nu_{S}+\mu_{S})(\rho +\varepsilon )}=\frac{2-3\alpha -2\beta }{2-\alpha -2\beta }\cdot \frac{\left(2-\alpha -2\beta )[\gamma_{S}(\rho +\varepsilon )+\theta \eta_{S}\right]}{2(\nu_{S}+\mu_{S})(\rho +\varepsilon )}\\ & =\zeta^{*}\cdot U_{S_{2}}^{*}>0 \end{array}$$ (69)

$$\begin{array}{cc} U_{R_{2}}^{*}-U_{R_{1}}^{*} & =\frac{\left(2-2\alpha -3\beta )[\gamma_{R}(\rho +\varepsilon )+\theta \eta_{R}\right]}{2(\omega_{R}+\lambda_{R})(\rho +\varepsilon )}=\frac{2-2\alpha -3\beta }{2-\alpha -2\beta }\cdot \frac{\left(2-2\alpha -\beta \right)[\gamma_{R}(\rho +\varepsilon )+\theta \eta_{R}]}{2(\omega_{R}+\lambda_{R})(\rho +\varepsilon )}\\ & =\varphi^{*}\cdot U_{R_{2}}^{*}>0 \end{array}$$ (70)

$$U_{S_{3}}^{*}-U_{S_{2}}^{*}=\frac{\left(\alpha +2\beta )[\gamma_{S}(\rho +\varepsilon )+\theta \eta_{S}\right]}{2(\nu_{S}+\mu_{S})(\rho +\varepsilon )}>0$$ (71)

$$U_{R_{3}}^{*}-U_{R_{2}}^{*}=\frac{\left(2\alpha +\beta )[\gamma_{R}(\rho +\varepsilon )+\theta \eta_{R}\right]}{2(\omega_{R}+\lambda_{R})(\rho +\varepsilon )}>0$$ (72)

$$U_{E_{3}}^{*}-U_{E_{2}}^{*}=\frac{\left(\alpha +\beta )[\gamma_{E}(\rho +\varepsilon )+\theta \eta_{E}\right]}{\left(\omega_{E}+\lambda_{E}+\nu_{E}+\mu_{E})(\rho +\varepsilon \right)}>0$$ (73)

**End of proof**.

**Proposition 2**: Proposition 2: The optimal returns to all three types of innovators in the cost-sharing scenario are more significant than in the no-incentive scenario, as in Eq (74).


$$V_{S_{2}}{\left(I\right)}^{*}\geq V_{S_{1}}{\left(I\right)}^{*},V_{R_{2}}{\left(I\right)}^{*}\geq V_{R_{1}}{\left(I\right)}^{*},V_{E_{2}}{\left(I\right)}^{*}\geq V_{E_{1}}{\left(I\right)}^{*}$$ (74)

**Proof**:

Eqs (24)–(26), (48)–(52) lead to Eqs (75)–(77).


$$V_{S_{2}}{\left(I\right)}^{*}-V_{S_{1}}{\left(I\right)}^{*}=\frac{\alpha (2-3\alpha -2\beta ){\left[\gamma_{S}(\rho +\varepsilon )+\theta \eta_{S}\right]}^{2}}{4\rho (\nu_{S}+\mu_{S}){\left(\rho +\varepsilon \right)}^{2}}+\frac{\alpha (2-2\alpha -3\beta ){\left[\gamma_{R}(\rho +\varepsilon )+\theta \eta_{R}\right]}^{2}}{2\rho (\omega_{R}+\lambda_{R}){\left(\rho +\varepsilon \right)}^{2}}\geq 0$$ (75)

$$V_{R_{2}}{\left(I\right)}^{*}-V_{R_{1}}{\left(I\right)}^{*}=\frac{\beta (2-3\alpha -2\beta ){\left[\gamma_{S}(\rho +\varepsilon )+\theta \eta_{S}\right]}^{2}}{2\rho (\nu_{S}+\mu_{S}){\left(\rho +\varepsilon \right)}^{2}}+\frac{\beta (2-2\alpha -3\beta ){\left[\gamma_{R}(\rho +\varepsilon )+\theta \eta_{R}\right]}^{2}}{4\rho (\omega_{R}+\lambda_{R}){\left(\rho +\varepsilon \right)}^{2}}\geq 0$$ (76)

$$V_{S_{2}}{\left(I\right)}^{*}-V_{S_{1}}{\left(I\right)}^{*}=\frac{{\left(3\alpha +2\beta -2\right)}^{2}{\left[\gamma_{S}(\rho +\varepsilon )+\theta \eta_{S}\right]}^{2}}{8\rho (\nu_{S}+\mu_{S}){\left(\rho +\varepsilon \right)}^{2}}+{\frac{{\left(2\alpha +3\beta -2\right)}^{2}[\gamma_{R}(\rho +\varepsilon )+\theta \eta_{R}]}{8\rho (\omega_{R}+\lambda_{R}){\left(\rho +\varepsilon \right)}^{2}}}^{2}\geq 0$$ (77)

**End of proof**.

**Proposition 3**: The optimal benefits of the green manufacturing innovation ecosystem are ranked in the three scenarios: the collaboration scenario is the largest, the cost-sharing scenario is the second largest, and the no-incentive scenario is the smallest, as in Eq (78).


$$V_{1}{\left(I\right)}^{*}<V_{2}{\left(I\right)}^{*}<V_{3}{\left(I\right)}^{*}$$ (78)

**Proof**:

Eqs (27), (48), (49), (53) and (64) lead to Eqs (79) and (80).


$$V_{2}{\left(I\right)}^{*}-V_{1}{\left(I\right)}^{*}=\frac{(3\alpha +2\beta -2)(\alpha -2\beta -2){\left[\gamma_{S}(\rho +\varepsilon )+\theta \eta_{S}\right]}^{2}}{8\rho (\nu_{S}+\mu_{S}){\left(\rho +\varepsilon \right)}^{2}}+{\frac{\left(2\alpha +3\beta -2)(-2\alpha +\beta -2)[\gamma_{R}(\rho +\varepsilon )+\theta \eta_{R}\right]}{8\rho (\omega_{R}+\lambda_{R}){\left(\rho +\varepsilon \right)}^{2}}}^{2}>0$$ (79)

$$V_{3}{\left(I\right)}^{*}-V_{2}{\left(I\right)}^{*}=\frac{{\left(\alpha +2\beta \right)}^{2}{\left[\gamma_{S}(\rho +\varepsilon )+\theta \eta_{S}\right]}^{2}}{8\rho (\nu_{S}+\mu_{S}){\left(\rho +\varepsilon \right)}^{2}}+{\frac{{\left(2\alpha +\beta \right)}^{2}[\gamma_{R}(\rho +\varepsilon )+\theta \eta_{R}]}{8\rho (\omega_{R}+\lambda_{R}){\left(\rho +\varepsilon \right)}^{2}}}^{2}+\frac{{\left(\alpha +\beta \right)}^{2}[\gamma_{E}(\rho +\varepsilon )+\theta \eta_{E}]}{2\rho (\omega_{E}+\lambda_{E}+\nu_{E}+\mu_{E}){\left(\rho +\varepsilon \right)}^{2}}>0$$ (80)

**End of proof**.

**Corollary 1**: the optimal incentive coefficient decreases with the increase of the return distribution coefficient, and the higher the return proportion distribution of the support layer and the scientific research layer, the smaller the optimal incentive coefficient of the application layer for the two types of subjects. Both types of subjects can obtain higher returns, and the application layer does not need to subsidize them heavily.

**Proof**:

Eq (81) can be obtained from Eqs (48) and (49).


$$\begin{array}{cc} \frac{\partial \zeta^{*}}{\partial \alpha }=-\frac{4(1-\beta )}{{\left(2-\alpha -2\beta \right)}^{2}}<0, & \frac{\partial \zeta^{*}}{\partial \beta }=-\frac{4\alpha }{{\left(2-\alpha -2\beta \right)}^{2}}<0,\\ \frac{\partial \varphi^{*}}{\partial \alpha }=-\frac{4\beta }{{\left(2-2\alpha -\beta \right)}^{2}}<0, & \frac{\partial \psi^{*}}{\partial \beta }=-\frac{4(1-\alpha )}{{\left(2-\alpha -2\beta \right)}^{2}}<0 \end{array}$$ (81)

**End of proof**.

**Proposition 4**: When the return distribution factors *α*, *β* satisfy the conditions of Eq (84), the collaborative scenario not only optimizes the overall returns of the green manufacturing innovation ecosystem but also enables the three types of subjects to achieve Pareto optimality.


$$g_{1}=\frac{{\left[\gamma_{S}(\rho +\varepsilon )+\theta \eta_{S}\right]}^{2}}{\rho (\nu_{S}+\mu_{S}){\left(\rho +\varepsilon \right)}^{2}},g_{2}=\frac{{\left[\gamma_{R}(\rho +\varepsilon )+\theta \eta_{R}\right]}^{2}}{\rho (\omega_{R}+\lambda_{R}){\left(\rho +\varepsilon \right)}^{2}},g_{3}=\frac{{\left[\gamma_{E}(\rho +\varepsilon )+\theta \eta_{E}\right]}^{2}}{\rho (\omega_{E}+\lambda_{E}+\nu_{E}+\mu_{E}){\left(\rho +\varepsilon \right)}^{2}}$$ (82)

$$\begin{array}{c} \frac{\alpha }{2}g_{1}+\frac{\alpha }{2}g_{2}+\frac{\alpha }{2}g_{3}\geq \frac{\alpha (2-\alpha -2\beta )}{4}g_{1}+\frac{\alpha (2-2\alpha -\beta )}{2}g_{2}+\alpha (1-\alpha -\beta )g_{3}\\ \frac{\beta }{2}g_{1}+\frac{\beta }{2}g_{2}+\frac{\beta }{2}g_{3}\geq \frac{\beta (2-\alpha -2\beta )}{2}g_{1}+\frac{\beta (2-2\alpha -\beta )}{4}g_{2}+\beta (1-\alpha -\beta )g_{3}\\ \frac{1-\alpha -\beta }{2}g_{1}+\frac{1-\alpha -\beta }{2}g_{2}+\frac{1-\alpha -\beta }{2}g_{3}\geq \frac{{\left(2-\alpha -2\beta \right)}^{2}}{8}g_{1}+\frac{{\left(2-2\alpha -\beta \right)}^{2}}{8}g_{2}+\frac{{\left(1-\alpha -\beta \right)}^{2}}{2}g_{3} \end{array}$$ (83)

The simplification leads to:

$$\begin{array}{c} g_{1}+g_{2}+g_{3}\geq \frac{\left(2-\alpha -2\beta \right)}{2}g_{1}+(2-2\alpha -\beta )g_{2}+2(1-\alpha -\beta )g_{3}\\ g_{1}+g_{2}+g_{3}\geq (2-\alpha -2\beta )g_{1}+\frac{\left(2-2\alpha -\beta \right)}{2}g_{2}+2(1-\alpha -\beta )g_{3}\\ g_{1}+g_{2}+g_{3}\geq \frac{{\left(2-\alpha -2\beta \right)}^{2}}{4(1-\alpha -\beta )}g_{1}+\frac{{\left(2-2\alpha -\beta \right)}^{2}}{4(1-\alpha -\beta )}g_{2}+(1-\alpha -\beta )g_{3} \end{array}$$ (84)

**Proposition 5**: When the initial innovation level a of *I*_0_ green manufacturing product is high and the effort level *U*_*i*_ and innovation capability *η*_*i*_ of the innovator are low, the cost of exploratory versus exploitative technology R&D causes the benefits of the innovation ecosystem to decline as the ecosystem evolves.

**Proof**:

Let Ω = *η*_*S*_*U*_*S*_ + *η*_*R*_*U*_*R*_ + *η*_*E*_*U*_*E*_, substituting it into Eq (2) leads to Eq (85).


$$I^{\prime }(t)=\Omega -\varepsilon I(t),I(t)=\frac{\Omega }{\varepsilon }+(I_{0}-\frac{\Omega }{\varepsilon })e^{-\varepsilon t}$$ (85)

According to Eqs (14), (38) and (58), it can be seen that the return function is a unitary function with *I* as the independent variable and *V*′(*I*) > 0. Hence, when $I_{0}-\frac{\Omega }{\varepsilon }>0$, $\frac{dV(I)}{dt}=V^{\prime }(I)\cdot I^{\prime }(t)=-\varepsilon V^{\prime }(I)(I_{0}-\frac{\Omega }{\varepsilon })e^{-\varepsilon t}<0$. That is, at $I_{0}>\frac{\eta_{S}U_{S}+\eta_{R}U_{R}+\eta_{P}U_{P}}{\varepsilon }$, the benefit *V* decreases gradually with ecosystem evolution.

**End of proof**.

## Numerical simulation and discussion

### Parameter assignment and solution

Based on the above model analysis, the optimal level of effort, the optimal return, and the optimal return of the innovation ecosystem as a whole of the three types of innovation subjects in different innovation equilibrium scenarios depend on the parameter assignment. Combining the parameter assignment logic of scholars [43] and referring to the indicator data on the green manufacturing industry in National Bureau of Statistics, the main variables are the number of innovation subjects such as industrial enterprises, universities, research institutes, and other variables represent the number of the application layer, the research layer, and the support layer; variables such as R&D expenditures of industrial enterprises represent the dual innovation balance costs of exploratory and exploitative innovation; and variables such as innovation technology facilities and industrial pollution inputs represent the green manufacturing environment. On this basis, the data are processed to simplify them to the same order of magnitude. The parameter assignments in this paper are shown in Table 2.

**Table 2:** Parameters and values.

parameter	Value	parameter	Value	parameter	Value	parameter	Value	parameter	Value
*I*(0)	0.05	ν_*S*_	0.5	ν_*E*_	0.3	*μ*_*S*_	0.5	*μ*_*E*_	0.3
*ω*_*R*_	0.4	*ω*_*E*_	0.2	*λ*_*R*_	0.4	*λ*_*E*_	0.2	*η*_*E*_	0.3
*η*_*S*_	0.4	*η*_*R*_	0.5	*γ*_*E*_	0.6	*γ*_*S*_	0.7	*γ*_*R*_	0.8
*θ*	0.3	*ρ*	0.1	*ε*	0.1	*α*	0.3	*β*	0.2

Based on the assignments in Table 2, the values of the variables in the three innovation balance scenarios are obtained by solving, and the plausibility of Propositions 1–5 is verified as shown below.

The no-incentive scenario:

$U_{S_{1}}^{*}=0.39$, $U_{R_{1}}^{*}=0.29$, $U_{E_{1}}^{*}=0.525$, $V_{S_{1}}{\left(I\right)}^{*}=4.2387$, $V_{R_{1}}{\left(I\right)}^{*}=2.7321$, $V_{E_{1}}{\left(I\right)}^{*}=6.9536$, *V*_1_(*I*)* = 13.9245, *V*_*S*1_ (*I*) = 4.2882 − 0.0495*e*^−0.1*t*^, *V*_*R*1_(*I*) = 2.7606 − 0.0285*e*^−0.1*t*^, *V*_*E*1_(*I*) = 7.0347 − 0.081*e*^−0.1*t*^, *V*_1_(*I*) = 14.544 − 0.6195*e*^-0.1*t*^.

The cost-sharing scenario:

$U_{S_{2}}^{*}=0.845$, $U_{R_{2}}^{*}=1.1625$, $U_{E_{2}}^{*}=0.525$, $V_{S_{2}}{\left(I\right)}^{*}=8.7297$, $V_{R_{2}}{\left(I\right)}^{*}=5.6677$, $V_{E_{2}}{\left(I\right)}^{*}=10.4288$, *V*_2_(*I*)* = 24.2374, *V*_*S*2_ (*I*) = 8.8592 − 0.1296*e*^−0.1*t*^, *V*_*R*2_(*I*) = 5.8271 − 0.1594*e*^−0.1*t*^, *V*_*E*2_(*I*) = 10.59 − 0.1988*e*^−0.1*t*^, *V*_2_(*I*) = 26.0139 − 1.7765*e*^-0.1*t*^.

The collaborative scenario:

$U_{S_{3}}^{*}=1.3$, $U_{R_{3}}^{*}=1.9375$, $U_{E_{3}}^{*}=1.05$, $V_{S_{3}}{\left(I\right)}^{*}=8.716$, $V_{R_{3}}{\left(I\right)}^{*}=5.8106$, $V_{E_{3}}{\left(I\right)}^{*}=14.5265$, *V*_3_(*I*)* = 29.0531, *V*_*S*3_ (*I*) = 8.9275 − 0.2115*e*^−0.1*t*^, *V*_*R*3_(*I*) = 6.0862 − 0.2756*e*^−0.1*t*^, *V*_*E*3_(*I*) = 14.725 − 0.1988*e*^−0.1*t*^, *V*_3_(*I*) = 31.6838 − 2.6307*e*^-0.1*t*^.

### Comparison of optimal returns for three scenarios

**Fig 3:**
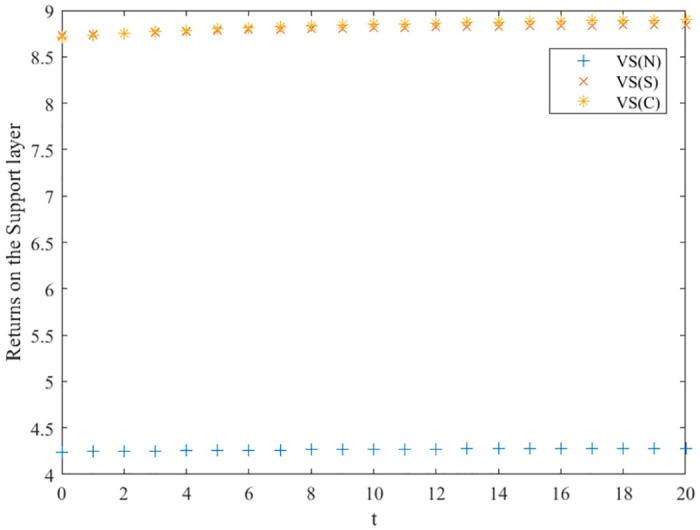
Optimal return of the support layer.

**Fig 4:**
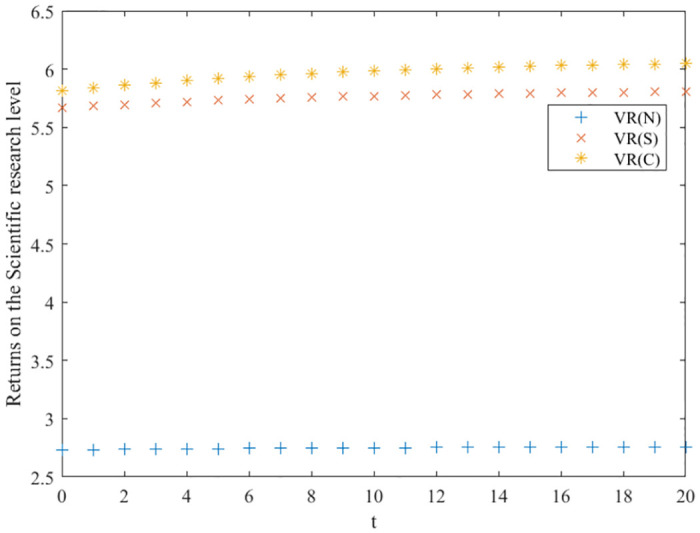
Optimal returns of the scientific research layer.

**Fig 5:**
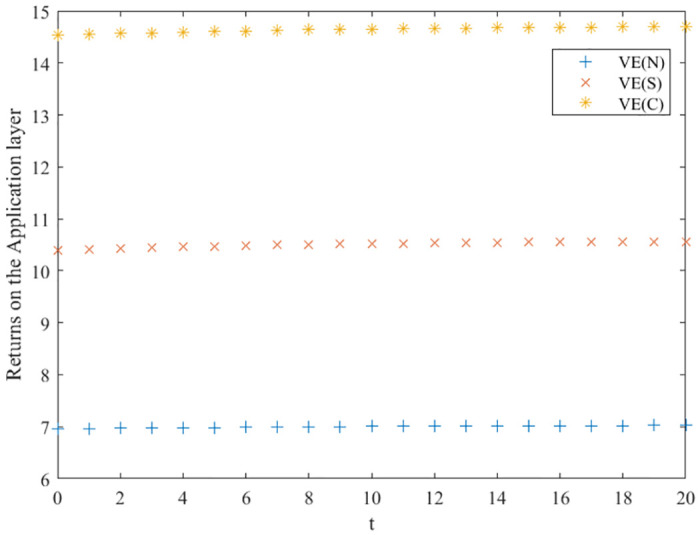
Optimal returns of the application layer.

**Fig 6:**
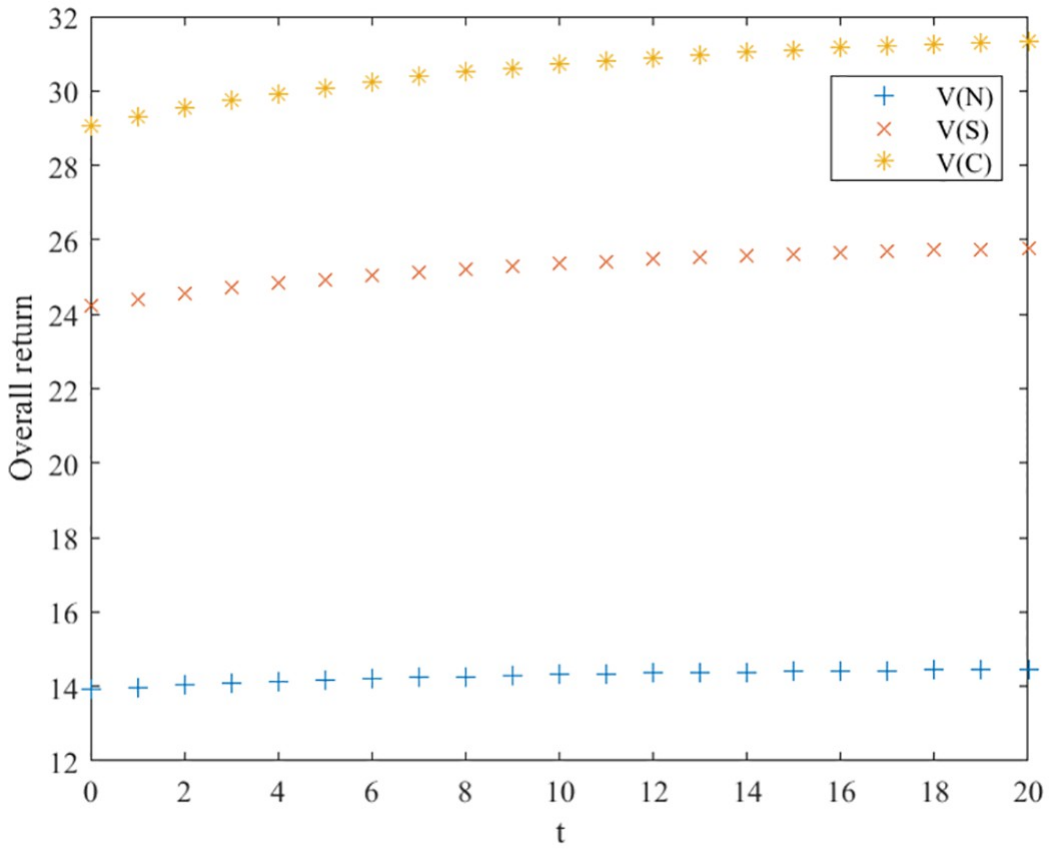
Optimal returns of the green manufacturing innovation ecosystem.

Based on the above analysis, the trend of the evolution of the three innovation subjects and the overall benefits of the green manufacturing innovation ecosystem in different innovation equilibrium game scenarios is derived, as shown in Figs 3–6, where the x-axis is the evolution time, and the y-axis is the subject’s benefits. We can conclude that in the collaborative and cost-sharing scenarios, the benefits of the three types of innovation subjects and the ecosystem increase over time, with a more significant increase in the early stage of evolution and then gradually converging to a stable state. Meanwhile, Figs 3–6 show that the collaborative scenario has the highest benefits, and the no-incentive scenario has the lowest benefits, consistent with Propositions 1–4.

In the no-incentive scenario, the returns of the three types of innovators do not increase significantly over time evolution and finally stabilize. Observing the return function and the optimal return function of the no incentive scenario, it can be seen that if the initial innovation level of green manufacturing products is high, the returns of the three types of innovation subjects will decrease over time; on the contrary, the returns of the three types of innovation subjects will gradually increase with the evolution of time. Green manufacturing innovation ecosystems embody eco-efficiency features, are rich in human resources, knowledge, and technological facilities, and the initial level of innovation of their products is relatively high. Hence, the cost-sharing and collaborative scenarios are more suitable for their development, and they can promote a higher-quality innovation balance.

### Impact of important variables in the collaborative scenario

#### Impact of exploratory and exploitative innovation cost factor (*ω*, *λ*)

**Fig 7:**
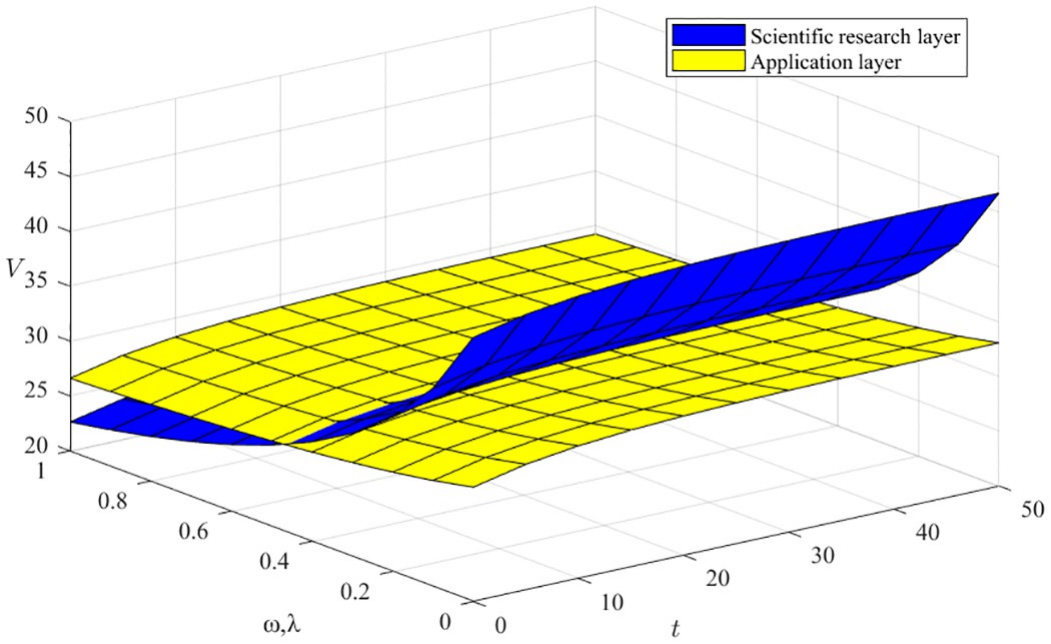
Impact of exploratory and exploitative innovation cost factor (*ω*, *λ*).

From Eq (64), $\frac{\partial V_{3}^{*}}{\partial \omega }<0$, $\frac{\partial V_{3}^{*}}{\partial \lambda }<0$ indicates that both exploratory and exploitative innovation capability coefficients negatively affect the benefits of green manufacturing innovation ecosystems, as shown in Fig 7, where the x-axis represents the evolution time, the y-axis represents the different exploratory and exploitative innovation balances, and the z-axis represents the benefits of the innovator. We can conclude that the impact of exploratory and exploitative innovations on ecosystem benefits is more significant in the early stage and gradually decreases in the later stage. This is because, in the early stage of ecosystem evolution, the dual innovation balance between exploratory and exploitative innovations is weak, and ecosystem development needs to be empowered.

#### Impact of the resource cost factor *ν* and the environmental construction cost factor *μ*

**Fig 8:**
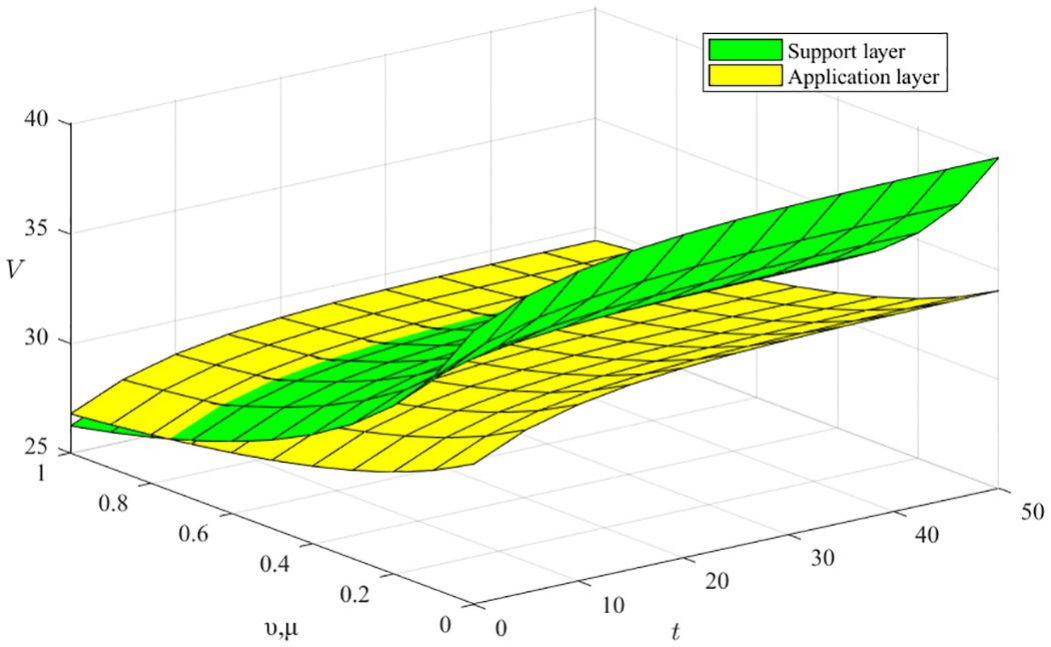
Impact of the resource cost factor *ν* and the environmental construction cost factor *μ*.

From Eq (64), $\frac{\partial V_{3}^{*}}{\partial \nu }<0$, $\frac{\partial V_{3}^{*}}{\partial \mu }<0$, the resource cost coefficient *ν* and the environmental construction cost coefficient *μ* both harm green manufacturing ecosystem benefits, as shown in Fig 8, where the x-axis represents the time evolution, the y-axis represents the different resource cost coefficients and environment building cost coefficients, and the z-axis represents the innovation subject benefits. We can conclude that the rate of decline in returns is faster when resource costs and environmental construction costs are lower, and the rate of decrease in returns gradually flattens out as the ecosystem evolves and both resource costs and environmental construction costs increase. Moreover, the effort of the support layer has an overall more significant impact on the revenue than that of the enterprise because the support layer is an essential carrier for the transformation of resource transfer and the optimization of environmental construction.

#### Impact of the innovation balance capacity factor *η*

**Fig 9:**
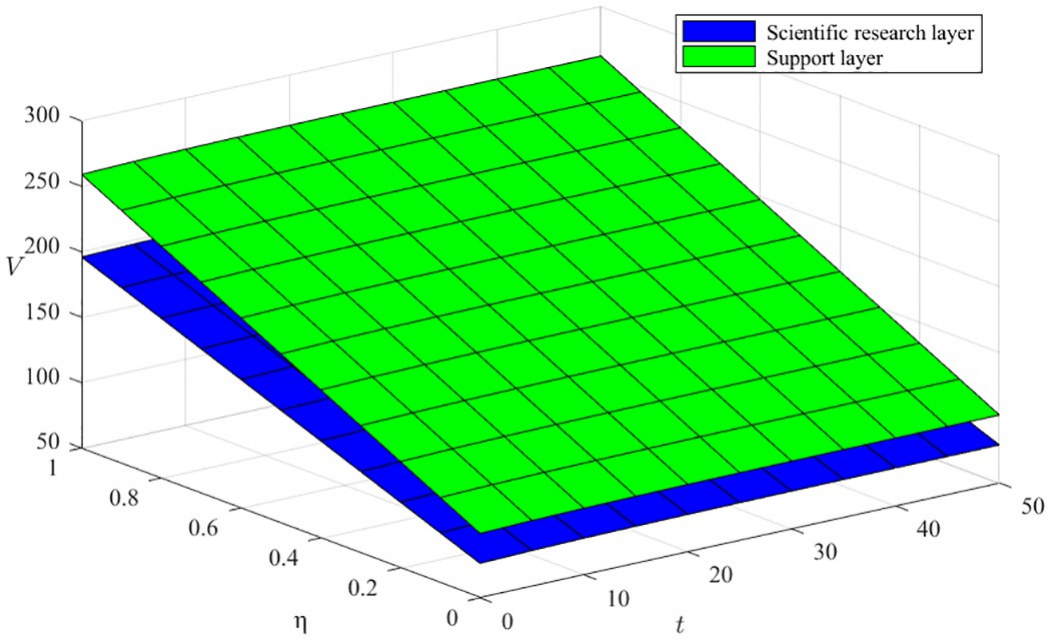
Impact of the innovation balance capacity factor on the support and scientific research layers.

**Fig 10:**
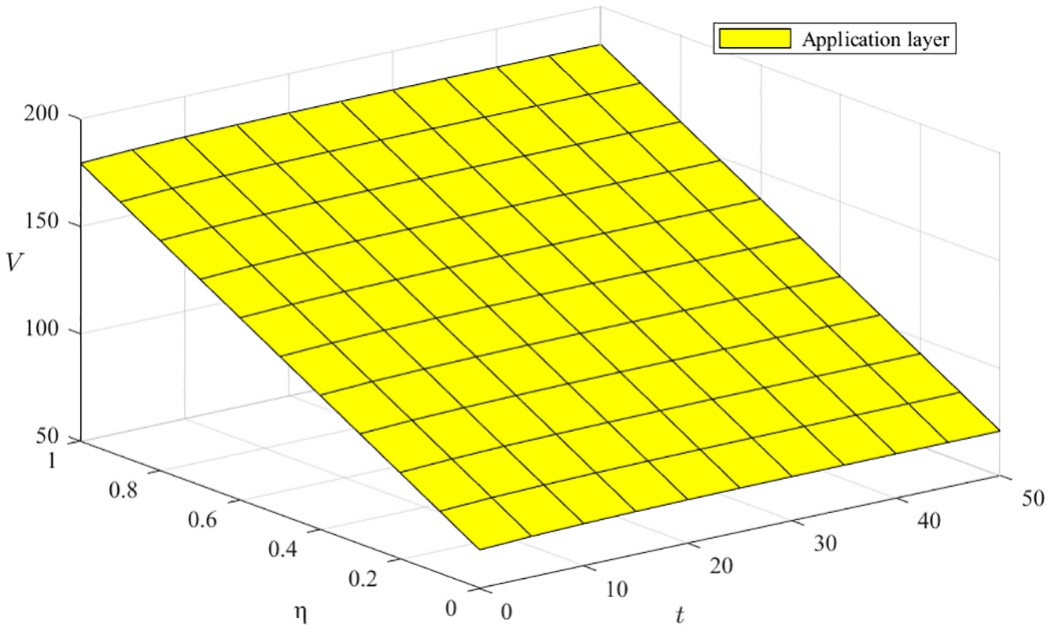
Impact of innovation balancing capacity factor on application layer.

From Eq (64), $\frac{\partial V_{3}^{*}}{\partial \eta }>0$, the coefficient of innovation equilibrium capacity a produces a positive benefit to the green manufacturing innovation ecosystem, as shown in Figs 9 and 10, where the x-axis represents the time evolution, the y-axis represents the different innovation balance coefficients, and the z-axis represents the innovation subject returns. We can conclude that the degree of effort of the three types of innovation subjects constantly impacts the rate of increase of benefits, indicating that high-quality innovation balancing ability can significantly enhance the overall benefits of the ecosystem. Moreover, the support layer plays a higher influence than the research layer, and the influence degree gradually increases with time evolution.

## Conclusions and implications

### Main conclusions

Taking the green manufacturing innovation ecosystem as the research object, this paper innovatively divides the main body into the application layer, scientific research layer, and support layer. It explores the dynamic evolution process of exploratory and exploitative innovation balance. Based on the reality, the collaboration mode of the three tiers of subjects is defined as three scenarios: no incentive, cost sharing, and cooperative, and the effort level and optimal benefit of the innovative subjects and the overall optimal benefit of the ecosystem are analyzed respectively, and the validity of the model is verified based on numerical simulation.

The results show that:

1. The evolutionary process of innovation balance affects the R&D decisions of innovation subjects, which affects the benefits of innovation subjects and ecosystems. Specifically, innovation balance capacity positively affects returns. In contrast, exploratory and exploitative innovation costs, R&D costs, and environmental construction costs negatively impact returns. Still, the innovation balance tends to converge to a high-quality balance as costs continue to be invested.
2. Among the three innovation balance scenarios, the synergistic symbiosis scenario is better than the no-incentive scenario when the green innovation conditions are consistent, and in the cost-sharing scenario, the cost subsidy factor changes in the opposite direction of the benefit distribution factor. When the support layer and the scientific research layer receive higher benefits, the application layer will reduce their subsidy.
3. The support layer’s gain over time could be more apparent, indicating that the support layer’s efforts are invested appropriately and consistently. Still, unreasonable inputs can produce a different degree of gain. The support layer should reasonably allocate resources and optimize the green manufacturing innovation environment to avoid being affected by the diminishing utility of scale.
4. When the initial level of green manufacturing products is high, and the innovator’s level of effort and innovation capacity is low, product development will lead to diminishing overall ecosystem benefits over time.

### Theoretical implications

1. Combining the innovation ecosystem theory with the green manufacturing theory deepens the research on green manufacturing from the perspective of complex systems. On the one hand, the core innovation subject in the green manufacturing innovation ecosystem is analyzed based on the hierarchical structure, which deepens the synergistic mechanism of different subjects in the innovation ecosystem [44] and responds to the scholars’ call for the use of the innovation ecosystem perspective to study the evolution of the green manufacturing field [45]. On the other hand, based on the impacts of multiple variables on the innovation subjects and ecosystem benefits, the differences in the strategic choices of the subjects at different levels in the innovation balance evolution scenarios are further identified, which provides an essential reference for the ecosystem to develop a high-quality innovation balance, responding to the scholars’ dissection of the influencing elements in the dynamic evolution process of the exploratory and exploitative innovation balances [46].
2. Introducing the differential game method into the evolution of dual innovation equilibrium in green manufacturing innovation ecosystems deepens the explanatory strength of dual innovation balance theory in innovation ecosystem research. This paper adopts the differential game approach to explore three scenarios in the evolution of innovation balance, providing a more granular understanding of ecosystem innovation balance. At the same time, the combination of numerical simulation intuitively analyzes the influence of essential variables on the evolution of innovation balance, which responds to the scholars’ call to promote the sustainable development of green manufacturing innovation ecosystems by adopting a new methodology to examine the evolution process and path of exploratory and exploitative innovation balance [47].

### Management implications

To better sustain the stable development of the green manufacturing innovation ecosystem and promote a balanced and high-quality development pattern of innovation, this paper draws the following management implications.

1. The three layers of innovation bodies are the primary carriers for driving the balance of innovation and should formulate appropriate innovation strategies based on their core functions. Facing the constraints of the innovation environment, enterprises should strengthen the connection with the support and research layers, take the initiative to bear a certain proportion of R&D costs for them, and actively explore collaboration with the outside bodies of the ecosystem.
2. Green manufacturing enterprises should continuously clarify their position in the innovation ecosystem through the dynamic evolution process of the balance between exploratory and exploitative innovation, and continue to expand and strengthen the ecosystem while benefiting from the win-win situation with other layers of innovators, aiming to reduce the rate of resource depletion and environmental pollution, and further contributing to the realization of the goal of carbon neutrality.
3. Local governments can aim at a high-quality innovation balance based on dual innovation theory for green innovation environment governance and system development. Relevant departments should formulate long-term planning, optimize the network construction of resource flow, capital flow and technology flow of innovation subjects, reduce the cost of resource absorption and integration, introduce human resources, investment and complementary innovation resources in a targeted manner [48], and formulate the ecosystem risk identification and prevention mechanism, to strive to achieve long-term, high-quality innovation balance and positive evolution.

## Research gaps and future prospects

This paper divides the innovation subjects in the green manufacturing innovation ecosystem into three layers. Still, there is a complex, competitive relationship within the subjects of the same layer, and to simplify the analysis and consider the space limitation, this paper does not model and analyze this issue. In addition to the incentives studied in this paper, there are also influencing factors such as technical standards, industrial alliances, standard features, user attraction, and retention [49]. Meanwhile, digital green innovation is becoming a new trend, and digital integration technology and digital networks have a facilitating effect on the technological capability enhancement of the innovation body, which is not explored in this paper either. Future research needs to broaden the research perspective and try to explore the optimal structure and subject relationship of the dual innovation balance of the green manufacturing innovation ecosystem, as well as the ways and means of digital technology’s empowerment of green manufacturing, to promote the innovation main body to continue to realize the balance of high-quality innovation in a stable state.
